# The human gut archaeome: identification of diverse haloarchaea in Korean subjects

**DOI:** 10.1186/s40168-020-00894-x

**Published:** 2020-08-04

**Authors:** Joon Yong Kim, Tae Woong Whon, Mi Young Lim, Yeon Bee Kim, Namhee Kim, Min-Sung Kwon, Juseok Kim, Se Hee Lee, Hak-Jong Choi, In-Hyun Nam, Won-Hyong Chung, Jung-Ha Kim, Jin-Woo Bae, Seong Woon Roh, Young-Do Nam

**Affiliations:** 1 Microbiology and Functionality Research Group, World Institute of Kimchi, Gwangju, 61755 Republic of Korea; 2 Research Group of Healthcare, Research Division of Food Functionality, Korea Food Research Institute, Jeollabuk-do, 55365 Republic of Korea; 3 Geologic Environment Division, Korea Institute of Geoscience and Mineral Resources, Daejeon, 34132 Republic of Korea; 4 Department of Family Medicine, Chung-Ang University Hospital, Chung-Ang University College of Medicine, Seoul, 06973 Republic of Korea; 5 Department of Biology, Kyung Hee University, Seoul, 02447 Republic of Korea

**Keywords:** Human gut, Population-level metataxonomic analysis, Archaeome, Haloarchaea, Archaeal enterotype

## Abstract

**Background:**

Archaea are one of the least-studied members of the gut-dwelling autochthonous microbiota. Few studies have reported the dominance of methanogens in the archaeal microbiome (archaeome) of the human gut, although limited information regarding the diversity and abundance of other archaeal phylotypes is available.

**Results:**

We surveyed the archaeome of faecal samples collected from 897 East Asian subjects living in South Korea. In total, 42.47% faecal samples were positive for archaeal colonisation; these were subsequently subjected to archaeal 16S rRNA gene deep sequencing and real-time quantitative polymerase chain reaction-based abundance estimation. The mean archaeal relative abundance was 10.24 ± 4.58% of the total bacterial and archaeal abundance. We observed extensive colonisation of haloarchaea (95.54%) in the archaea-positive faecal samples, with 9.63% mean relative abundance in archaeal communities. Haloarchaea were relatively more abundant than methanogens in some samples. The presence of haloarchaea was also verified by fluorescence in situ hybridisation analysis. Owing to large inter-individual variations, we categorised the human gut archaeome into four archaeal enterotypes.

**Conclusions:**

The study demonstrated that the human gut archaeome is indigenous, responsive, and functional, expanding our understanding of the archaeal signature in the gut of human individuals.

Video Abstract

## Background

The human gut harbours various biological entities such as bacteria, archaea, unicellular eukaryotes, and viruses [1]. These microbial entities constitutively contribute to the microbial signature, thereby maintaining the inherent characteristics of the gastrointestinal tract. To date, studies have focused on the genetic and functional traits of gut bacteria [2]. The introduction of next-generation sequencing technologies in gut microbiology has revealed the identity of gut microorganisms. Metagenomics-based gut microbial surveys on healthy populations [3, 4], as well as on those with various illnesses [5], have shed light on the characteristics of the bacterial microbiome under both eubiotic and dysbiotic conditions. The rigid basic information regarding the natural members of the bacterial microbiota has prompted further studies on the function of these microorganisms.

The domain Archaea was proposed as a separate group of prokaryotes in 1990 based on the ribosomal RNA gene sequences. Although most archaea are thought to be extremophiles, living in harsh environments, mesophilic archaea have been identified in moderate environments, such as the soil and ocean [6, 7]. Additionally, several studies have confirmed the presence of archaea on the human skin [8] and in the mouth [9] and gut [10, 11]. Although they are relatively minor, archaea are an important component of the human microbiome [12], and thus might have a complex community composition and structure on various human body sites.

Information regarding the community-based genetic and functional traits of archaea in animal habitats is scarce. We have previously reported occurrences of diverse members of extremely halophilic archaea (haloarchaea) in avian plumage [13], as well as in food samples such as salt-fermented seafood [14] and solar salts [15, 16]. In the human gut where the microbial entities thrive more abundantly than in other parts of the human body, the archaeome consisted mostly of methane-producing archaea (methanogens), of which, members belonging to the orders *Methanobacteriales* (including *Methanobrevibacter smithii* and *Methanosphaera stadtmanae*) and *Methanomassiliicoccales* (including *Methanomethylophilaceae*) are predominant [17]. However, studies using culture-independent approaches have reported that not all archaea in the human gut are methanogens. For instance, our previous study was the first to report the presence of haloarchaea in faecal samples of Korean subjects in 2008 based on a conventional molecular ecology method [18]. More recently, viable haloarchaeal strains (belonging to the genus *Haloferax*) were isolated from human faeces [19, 20], and two genome sequences of the human gut-derived haloarchaeal strains (i.e. *Haloferax massiliensis* and *Halorubrum lipolyticum*) are currently available [21]. However, the presence of the haloarchaeal community and their collective genomes (hereafter termed ‘haloarchaeome’) in the human gut was not confirmed despite the use of metataxonomic analysis, i.e. the archaeal 16S rRNA gene-targeted amplicon sequencing. In addition, archaeal members from the orders *Sulfolobales* and *Nitrososphaerales* have been detected in the human gut [17]. These observations suggest that the diversity and/or abundance of the human gut archaeome may vary with host factors, including diet and age. Methodological pitfalls (such as the selection of primer pairs and the sequence processing pipeline used) may also contribute to the low resolution of the human gut archaeome [22]. Collectively, there is no sufficient information on the identity of archaea in the gut environment.

In this study, we conducted a population-level metataxonomic analysis of the human gut archaeome. First, we screened 897 faecal samples collected from a cohort of Koreans, of which, 381 archaea-positive faecal samples were subjected to deep sequencing of the 16S rRNA gene amplicons and real-time quantitative polymerase chain reaction (PCR)-based abundance estimation, as well as Fluorescence in situ hybridisation (FISH) analysis. The Korean gut archaeome featured large inter-individual variation. We categorised the human gut archaeome into four archaeal enterotypes, i.e. the *Methanobacteriaceae*-, *Methanomethylophilaceae*-, *Haloferacaceae*- and the unclassified *Euryarchaeota*-dominated archaeome. We further assessed the correlation between the host metadata (dietary nutrients, food categories and clinical phenotypes) and the abundances of archaeal taxa to evaluate the host factors affecting the community structure of the human gut archaeome. Overall, we have attempted to understand the archaeal signature in the human gut.

## Results

### Extensive profiling of the Korean gut archaeome

This study includes faecal samples collected from 897 East Asian subjects living in South Korea. Although the detection of archaea is highly dependent on the methodology used, a maximum prevalence of 23% and 97.5% has been reported for the methanogens belonging to the order *Methanobacteriales*: *Methanobrevibacter smithii* and *Methanosphaera stadtmanae*, respectively, in the human gastrointestinal tract [12]. We first determined the presence of archaeal colonisation in all samples using an archaea-specific primer set. The results showed that 381 out of 897 faecal samples (42.47%) were positive for archaeal colonisation, and the positive samples were subsequently subjected to deep sequencing of the gut archaeome. In total, 275,909,328 reads were obtained from the Illumina Hiseq^TM^ X platform. After quality control, the remaining 193,370,457 reads (mean: 507,534 reads per sample; median: 240,156) were subjected to further analysis. The rarefaction analysis based on observed amplicon sequence variants (ASVs) showed that the sequencing depth had reached saturation (see Additional file 1: Supplementary Fig. S1a). Annotation of the archaeal 16S rRNA gene sequences to the SILVA database led to the prediction of 685 ASVs in the Korean gut archaeome (see Additional file 1: Supplementary Fig. S2).

**Fig. 1:**
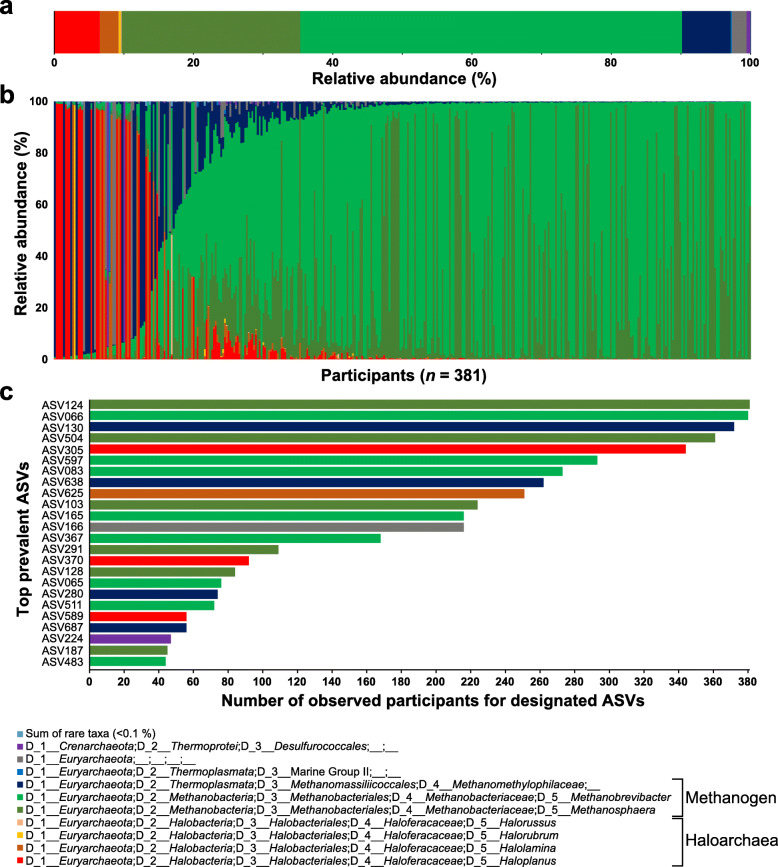
Profiles of the Korean gut archaeome. The archaeal 16S rRNA gene amplicons were prepared from faecal samples collected from the 381 Koreans, subjected to Illumina sequencing, and taxonomically assigned to the SILVA v. 132 database. **a** The community structure of the total human gut archaeome is shown as the mean relative abundance at the genus level. **b** The relative abundances of the gut archaeome are shown individually at the genus level, arranged in ascending order based on the genus *Methanobrevibacter*. **c** Prevalence of abundant taxa (> 0.1% of relative abundance) in all faecal samples. Amplicon sequence variants (ASVs) observed for > 10% of the participants were included. The ASVs are coloured according to their genus level and classified using the SILVA v. 132 database

The taxonomic classification of the gut archaeome revealed the predominance of sequences assigned to the phylum *Euryarchaeota*, followed by the phylum *Crenarchaeota* (Fig. 1a, b). At the genus level, the Korean gut archaeome showed proportionally abundant sequences assigned to the methanogen group; the genera *Methanobrevibacter* and *Methanosphaera* in the family *Methanobacteriaceae* of the order *Methanobacteriales* were mostly proportionally abundant (54.89% and 25.68% relative abundance, respectively) with minor contributions from the unclassified *Methanomethylophilaceae*. In particular, the Korean gut archaeome contained haloarchaea-assigned sequences with 9.63% mean relative abundance; sequences belonging to the genera *Halolamina*, *Haloplanus*, *Halorubrum*, *Halobacterium*, *Haloterrigena*, *Natronomonas*, *Halarchaeum*, *Haloarcula*, *Halonotius* and *Halorussus* were also detected. At the individual level, certain participants harboured the haloarchaea-dominated archaeal community structure (i.e. haloarchaea-assigned sequences showed 99.33% relative abundance; Fig. 1b). Next, we assessed the core ASVs, i.e. ASVs detected extensively in all faecal samples. The methanogen-assigned ASVs (e.g. ASV124, ASV066 and ASV130) were detected in > 97% of the total samples. In contrast, haloarchaea-assigned ASVs (e.g. ASV305 assigned to the genus *Haloplanus*) were detected in 95.54% of the archaea-positive samples (Fig. 1c), suggesting that members belonging to both methanogens and haloarchaea might be the soft core microbial component, i.e. over 95% detection rate but not 100%.

### Abundance estimation of the human gut archaea

**Fig. 2:**
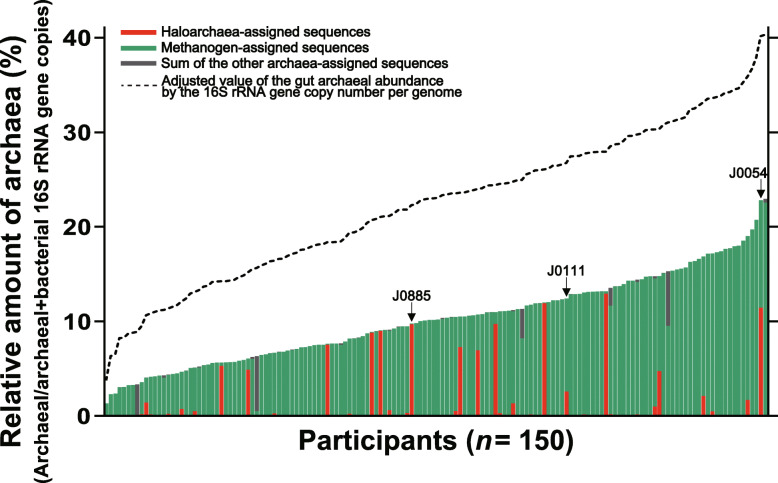
Quantitative analysis of the human gut archaea. The archaeal 16S rRNA gene copy number was quantified from faecal samples of Korean subjects using real-time quantitative PCR (*n* = 150, three technical replicates). Values were normalised to the abundance of the archaeal + bacterial 16S rRNA genes and are presented as relative amounts. The amount of the archaeal + bacterial 16S rRNA genes in each sample was arbitrarily considered 100. Colours of each bar graph represent the relative abundance from the archaeal 16S rRNA gene metataxonomic data. The dotted line represents the adjusted value of the gut archaeal abundance by the 16S rRNA gene copy number per genome (a mean of 1.7 and 5.0 16S rRNA genes per archaeal and bacterial genome, respectively)

The abundances of bacterial cells (10^9^ to 10^11^ bacteria g^−1^ faeces) in the human gut have been well described [23, 24]. We attempted to estimate the archaeal abundance by determining the archaea/(archaea + bacteria) ratios. We randomly selected 150 samples from the 381 archaeome-positive faecal samples and quantified both the archaeal and bacterial 16S rRNA gene copy numbers using real-time quantitative PCR. The results showed that the archaeal abundance was 10.24 ± 4.58% (mean ± standard deviation, SD) of the total bacterial and archaeal abundance (Fig. 2). The currently available information suggests that archaea and bacteria possess a mean of 1.7 and 5.0 16S rRNA genes per genome, respectively (https://rrndb.umms.med.umich.edu/ [25]). Based on this, we attempted to correct the gut archaeal abundance by the number of 16S rRNA genes. As shown in Fig. 2, the adjusted archaeal abundance accounted for 22.35 ± 7.90% (mean ± SD) of the total bacterial and archaeal abundance.

### Compositional characterisation of the Korean gut archaeome

**Fig. 3:**
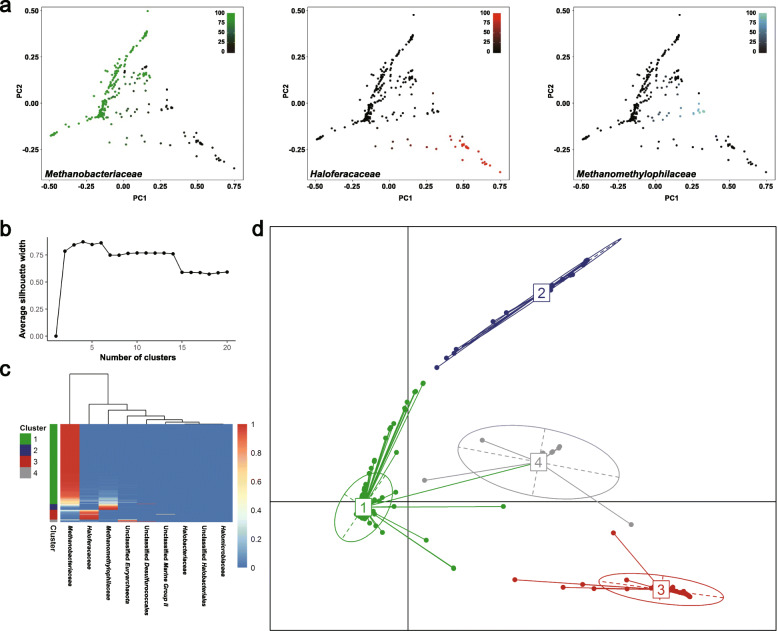
Identification of enterotypes in the Korean gut archaeome. The faecal archaeal 16S rRNA gene sequences were prepared from Korean individuals. We subsampled 342 from the 381 archaeal-sequence–positive samples at a sampling depth of 10,000. **a** PCoA was generated based on the weighted UniFrac distance matrix of the archaeal 16S rRNA gene sequence data. Colour gradations show the relative abundances of the families *Methanobacteriaceae* (green), *Haloferacaceae* (red) and *Methanomethylophilaceae* (blue). **b**–**d**. Assortment of gut archaeal communities into enterotypes. **b** The optimal number of clusters was estimated at the family level by partitioning around medoids (PAM) clustering based on the Bray-Curtis dissimilarity matrix. **c** The relative abundance of the archaeal communities in all samples are presented according to the clusters and shown as a heat map. **d** Visualisation of enterotypes. The dots and numbers represent the abundance distributions of the archaeal taxa from individual samples and the centre of each enterotype, respectively

One of the major features of the human bacterial microbiome is inter-individual variation commonly observed even in healthy individuals [26]. Our metataxonomic analysis revealed remarkable differences in the archaeal structure of individual Korean guts (Fig. 1). Therefore, we assessed the compositional features of the gut archaeome of 342 subsampled individuals (i.e. the number of sequences was evenly normalised at a sampling depth of 10,000 across the subjects; see Additional file 1: Supplementary Fig. S1b). Principal coordinate analysis (PCoA) of the weighted UniFrac distance matrix revealed that the per cent relative abundances of several key archaeal taxa, i.e. families *Methanobacteriaceae*, *Haloferacaceae* and *Methanomethylophilaceae*, are the discriminant factors determining the distance between samples (Fig. 3a).

The gut bacterial communities in the human gastrointestinal tract have been partitioned into several clusters. Each cluster (enterotype) is overrepresented by a distinct set of bacterial genera [27]. As shown in the taxonomic and clustering analyses above, the community composition and structure of the Korean gut archaeome highlighted the importance of the several robust clusters that were prevalent across samples with different abundances. Next, we assessed the presence of enterotypes by applying partitioning around medoids (PAM) clustering analysis to the Bray-Curtis dissimilarity matrix generated from the family-level relative abundance profiles (Fig. 3b). The results showed four distinct clusters: *Methanobacteriaceae* as archaeal enterotype (MBA enterotype), *Methanomethylophilaceae* as archaeal enterotype (MMA enterotype), *Haloferacaceae* as archaeal enterotype (HFA enterotype) and the unclassified *Euryarchaeota* related to uncultured phylotypes in genus *Methanosphaera* or *Haloplanus* as archaeal enterotype (UEA enterotype; Fig. 3c, d). These suggested that the distinct set of overrepresented taxa (enterotypes) might be the consequence of a well-balanced symbiotic relationship between the host and archaea in the gut environment.

### Diversity of haloarchaeal phylotypes in the human gut

**Fig. 4:**
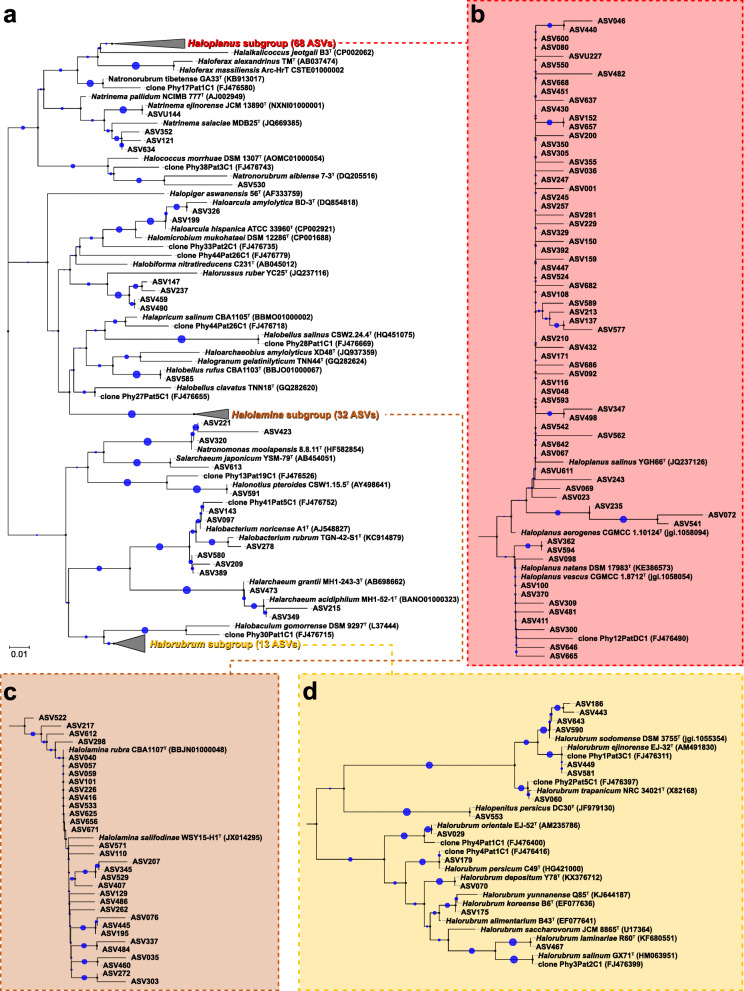
Phylogenetic analysis of the amplicon sequence variants (ASVs) of haloarchaea. The ASVs assigned to the haloarchaea were identified in the faecal archaeal 16S rRNA gene sequence data of the 381 Koreans. The 16S rRNA gene sequences of the validated haloarchaeal species and public clonal sequences were included. **a** A phylogenetic consensus tree based on the 16S rRNA gene sequences was reconstructed using the neighbour joining algorithm, indicating the taxonomic positions of the ASVs. **b**–**d** Phylogenetic trees of the three subgroups are shown: *Haloplanus* subgroup (**b**), *Halolamina* subgroup (**c**) and *Halorubrum* subgroup (**d**). The size of blue circles represents the bootstrap value based on 1000 replications. Bar, 0.01 accumulated changes per nucleotide

Phylogenetic analysis of the methanogen-assigned sequences revealed three clusters of the methanogen phylotypes with the genera *Methanobrevibacter* (318 ASVs) and *Methanosphaera* groups (178 ASVs) and the family *Methanomethylophilaceae* group (44 ASVs; see Additional file 1: Supplementary Fig. S3). Studies on human gut archaeomes dominated by methanogens have been already described elsewhere [12, 17, 22, 28]. Therefore, we analysed the haloarchaea-assigned sequences, which have not yet been reported in the metataxonomic studies of the human gut archaeome. To evaluate the diversity of the haloarchaeal phylotypes in the human gut, we conducted a phylogenetic analysis based on the 139 haloarchaea-assigned ASVs with trimmed 16S rRNA gene sequences of the validated haloarchaeal species and public clonal sequences. We observed that the majority of haloarchaeal phylotypes are closely related to the genera *Haloplanus*, *Halolamina* and *Halorubrum* (Fig. 4a). In particular, we observed three branched clusters of the haloarchaeal phylotypes, which were relatively distantly located from the validated haloarchaeal species. The *Haloplanus* subgroup consisted of 68 ASVs (Fig. 4b); *Halolamina*, 32 ASVs (Fig. 4c); and *Halorubrum*, 13 ASVs (Fig. 4d). We next assessed whether sequences assigned to haloarchaea occur in other sample cohorts by trawling the publicly available human metagenomic and metataxonomic datasets bases using the EBI MGnify. As shown in Table 1, we found several studies possessing the metagenomic and metataxonomic sequences assigned to haloarchaea, implying that the human gut is capable of harbouring diverse and metabolically unknown haloarchaeal strains.

**Table 1:** Haloarchaea-assigned sequences in other publicly available metagenomic and metataxonomic studies

Accession number	Study type	Amplified region	Study name	Haloarchaea-assigned taxon
ERP001956	Metagenomic	N/A	Diagnostic Metagenomics: A Culture-Independent Approach to the Investigation of Bacterial Infections	c__Halobacteria; o__Halobacteriales; f__Halobacteriaceae
SRP001634	Metagenomic	N/A	Microbial composition of samples from infant gut	c__Halobacteria; o__Halobacteriales; f__Halobacteriaceae; g__; s__ c__Halobacteria; o__Halobacteriales; f__Halobacteriaceae; g__Halococcus; s__ c__Halobacteria; o__Halobacteriales; f__Halobacteriaceae; g__Halorhabdus; s__
SRP073172	Metagenomic	N/A	DNA from FIT can replace stool for microbiota-based colorectal	c__Halobacteria; o__Halobacteriales; f__Halobacteriaceae; g__; s__
SRP096283	Metagenomic	N/A	Human gut metagenome and metatranscriptome raw sequence reads	c__Halobacteria
SRP118697	Metagenomic	N/A	Microbiome and Worm Infection	c__Halobacteria; o__Halobacteriales
SRP128128	Metagenomic	N/A	Dynamics of human gut microbiota and metabolites in response to prebiotic interventions	c__Halobacteria c__Halobacteria; o__Halobacteriales c__Halobacteria; o__Halobacteriales; f__Halobacteriaceae; g__Halobacterium c__Halobacteria; o__Halobacteriales; f__Halococcaceae; g__Halococcus c__Halobacteria; o__Natrialbales; f__Natrialbaceae; g__Natronococcus
ERP015450	Metagenomic	N/A	Dysbiosis of gut microbiota contributes to the pathogenesis of hypertension	c__Halobacteria; o__Halobacteriales; f__Halobacteriaceae; g__Haloarcula; s__
ERP005883	Metataxonomic	V4	Effects of cholera on the human gut microbiota, and interactions between human gut microbes and Vibrio cholera	c__Halobacteria; o__Halobacteriales; f__Halobacteriaceae; g__; s__
				c__Halobacteria; o__Halobacteriales; f__Halobacteriaceae; g__Halorubrum; s__
				c__Halobacteria; o__Halobacteriales; f__Halobacteriaceae; g__Haloterrigena; s__
ERP010229	Metataxonomic	V4	Gut microbial succession follows acute secretory diarrhea in humans	c__Halobacteria; o__Halobacteriales; f__Halobacteriaceae; g__; s__
				c__Halobacteria; o__Halobacteriales; f__Halobacteriaceae; g__Halalkalicoccus; s__jeotgali
				c__Halobacteria; o__Halobacteriales; f__Halobacteriaceae; g__Halococcus; s__
				c__Halobacteria; o__Halobacteriales; f__Halobacteriaceae; g__Halococcus; s__hamelinensis
				c__Halobacteria; o__Halobacteriales; f__Halobacteriaceae; g__Halorhabdus; s__
				c__Halobacteria; o__Halobacteriales; f__Halobacteriaceae; g__Haloterrigena; s__
				c__Halobacteria; o__Halobacteriales; f__Halobacteriaceae; g__Natronococcus; s__
ERP021093	Metataxonomic	V4	Gut microbiome from patients obtained by 16s rRNA sequencing	c__Halobacteria; o__Halobacteriales; f__Halobacteriaceae; g__; s__
				c__Halobacteria; o__Halobacteriales; f__Halobacteriaceae; g__GA41; s__
				c__Halobacteria; o__Halobacteriales; f__Halobacteriaceae; g__Haloarcula; s__
				c__Halobacteria; o__Halobacteriales; f__Halobacteriaceae; g__Halobacteriaceae; s__GX3
				c__Halobacteria; o__Halobacteriales; f__Halobacteriaceae; g__Halobacterium; s__
				c__Halobacteria; o__Halobacteriales; f__Halobacteriaceae; g__Halococcus; s__
				c__Halobacteria; o__Halobacteriales; f__Halobacteriaceae; g__Halomicrobium; s__
				c__Halobacteria; o__Halobacteriales; f__Halobacteriaceae; g__Halomicrobium; s__mukohataei
				c__Halobacteria; o__Halobacteriales; f__Halobacteriaceae; g__Halonotius; s__
				c__Halobacteria; o__Halobacteriales; f__Halobacteriaceae; g__Haloplanus; s__
				c__Halobacteria; o__Halobacteriales; f__Halobacteriaceae; g__Haloquadratum; s__
				c__Halobacteria; o__Halobacteriales; f__Halobacteriaceae; g__Halorhabdus; s__
				c__Halobacteria; o__Halobacteriales; f__Halobacteriaceae; g__Halorubrum; s__
				c__Halobacteria; o__Halobacteriales; f__Halobacteriaceae; g__Natronomonas; s__
				c__Halobacteria; o__Halobacteriales; f__Halobacteriaceae; g__XKL75; s__
				c__Halobacteria; o__Halobacteriales; f__MSP41; g__; s__
ERP021896	Metataxonomic	V4	Moving pictures of the human microbiome	c__Halobacteria; o__Halobacteriales
				c__Halobacteria; o__Halobacteriales; f__Halobacteriaceae
				c__Halobacteria; o__Halobacteriales; f__Halobacteriaceae; g__Halarchaeum
				c__Halobacteria; o__Halobacteriales; f__Halobacteriaceae; g__Halobacterium
ERP107577	Metataxonomic	V3orV4	LogMPIE: Landscape Of Gut Microbiome - Pan India Exploration	c__Halobacteria
				c__Halobacteria; o__Haloferacales
				c__Halobacteria; o__Haloferacales; f__Haloferacaceae
				c__Halobacteria; o__Haloferacales; f__Halorubraceae; g__Haloparvum
				c__Halobacteria; o__Natrialbales; f__Natrialbaceae
ERP109659	Metataxonomic	V3-V4	Gut microbiota in Parkinson's disease: temporal stability and disease progression	c__Halobacteria; o__Halobacteriales

*N*/*A* not applicable

A positive correlation between the richness of the halophilic bacteria and faecal salinity has been recently reported [19]. To verify this association, we randomly selected 20 faecal samples with different percent relative abundances of haloarchaea, i.e. 5.38–99.33%, and measured faecal salinity using a salinity refractometer, yielding a mean salinity of 0.68%, ranging from 0.30 to 1.05% (see Additional file 1: Supplementary Fig. S4a). Correlation coefficient and linear regression analyses revealed no association between the relative abundance of haloarchaea and faecal salinity. Further, we analysed the inorganic elements mainly consisting of the salt (e.g. sodium, potassium, magnesium and calcium) of 20 selected samples using an inductively coupled plasma-mass spectrometer (ICP-MS). On average, each inorganic element possessed less than 1% of total faecal weight: 0.04%, 0.40%, 0.24% and 0.50% for sodium, potassium, magnesium and calcium, respectively (see Additional file 1: Supplementary Fig. S4b). Similar to the salinity data, no positive or negative relationship was found between the relative abundance of haloarchaea and the relative amount of faecal inorganic elements.

### Detection of haloarchaea in the human gut by fluorescence in situ hybridisation

**Fig. 5:**
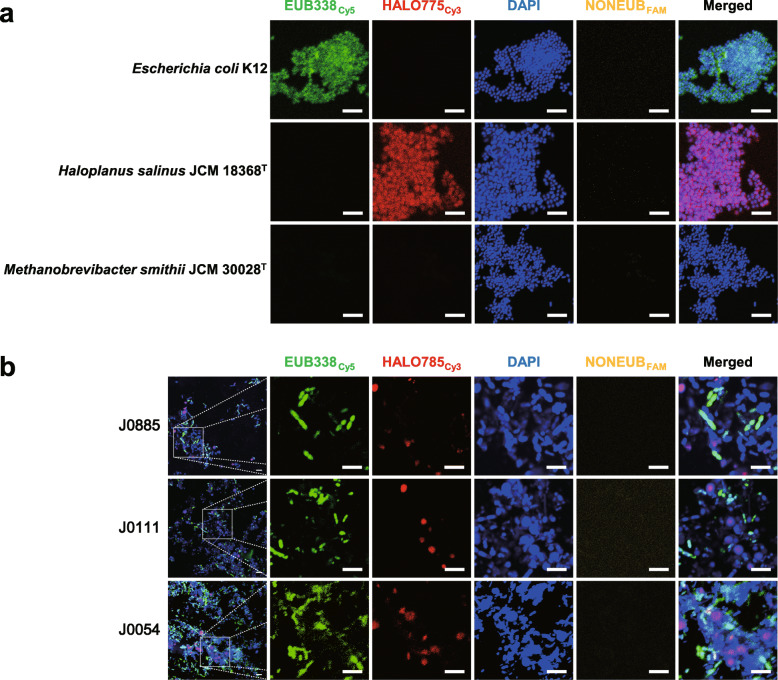
Detection of the gut haloarchaea by fluorescence *in situ* hybridisation (FISH). **a** The binding specificity of the haloarchaea-specific probe HALO775_Cy3_ (red) to a cultured bacterium (*Escherichia coli* K12), haloarchaeon (*Haloplanus salinus* JCM 18368^T^) and other archaeon (*Methanobrevibacter smithii* JCM 30028^T^) were tested. A probe EUB338_Cy5_ (green) was used for detection of bacteria. A counterstain was performed using DNA staining solution (4′,6-diamidino-2-phenylindole, DAPI; blue). **b** To verify the presence of haloarchaea in the human gut, three faecal samples (J0885, J0111 and J0054) were subjected to FISH analysis. Non-binding probe (NONEUB_FAM_) was included to decipher false positives. Scale bars correspond to 5 μm

We attempted to verify the presence of haloarchaea in the human gut by a non-sequencing-based approach using the FISH analysis. We designed a haloarchaea-specific probe (HALO775) and tested the specificity of the oligonucleotide probe. Neither the undesired match (i.e. in silico binding of the HALO775 with non-haloarchaeal taxa, such as methanogen, bacteria and eukaryotes; see Additional file 2: Supplementary Tables S1 and S2) nor the unspecific binding of the HALO775_cy3_ with the cultured bacterium (*Escherichia coli* K12) and other archaeon (*Methanobrevibacter smithii* JCM 30028^T^) was found (Fig. 5a). A positive signal of the HALO775_cy3_ was only observed with the cultured haloarchaeon (*Haloplanus salinus* JCM 18368^T^). Based on both the total archaeal abundance and the metataxonomic data, we selected three faecal samples that possess a different proportion of the haloarchaea-assigned sequences (sample J0885, J0111 and J0054; Fig. 2). The FISH analysis successfully detected a positive signal for haloarchaea from the selected samples (Fig. 5b). These results collectively suggest the presence of haloarchaea in the human gut, which was detected by sequencing-based (i.e. metataxonomics) and non-sequencing-based (i.e. FISH) methods.

### Correlation analysis of the gut archaeal profiles with host factors

**Fig. 6:**
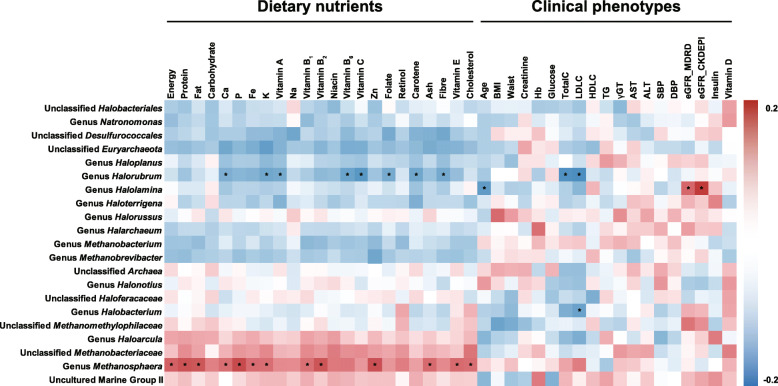
Correlation analysis of the gut archaeal taxa and host factors. Spearman’s rank correlation coefficients and the corresponding *P* values were calculated based on comparisons of the relative abundance of abundant archaeal taxa at the genus level and the host factors, including dietary nutrients and clinical phenotypes. For orphan sequences (i.e. unclassified at the genus level), a high-rank lineage is provided. *BMI* body mass index, *Hb* haemoglobin, *TotalC* total cholesterol, *LDLC* low-density lipoprotein cholesterol, *HDLC* high-density lipoprotein cholesterol, *TG* triglyceride, *γGT* gamma-glutamyltransferase, *AST* aspartate transaminase, *ALT* alanine transaminase, *SBP* systolic blood pressure, *DBP* diastolic blood pressure, *eGFR*_*MDRD* estimated glomerular filtration rate using the MDRD formula, *eGFR*_*CKDEPI* estimated glomerular filtration rate using the CKD-EPI formula. Correction for multiple comparisons used the false discovery rate (FDR; threshold of 0.05). *Adjusted *P* < 0.05

We next evaluated the effect of host factors on the community structure of the Korean gut archaeome by conducting correlation coefficient analysis using two variables: relative abundance of the proportionally abundant archaeal taxa at the genus level and host factors, including dietary nutrients, clinical phenotypes and food categories. We observed strong negative/positive correlations (Spearman's rank correlation analysis, adjusted *P* < 0.05) between several archaeal taxa and dietary nutrients: the genus *Halorubrum* was negatively correlated with calcium, potassium, vitamin A, vitamin B_6_, vitamin C, folate, carotene and fibre levels; the genus *Methanosphaera* was positively correlated with energy, protein, fat, phosphorus, iron, potassium, vitamin B_1_, vitamin B_2,_ zinc, ash, vitamin E and cholesterol levels (Fig. 6 left). In host clinical phenotypes, the genera *Halorubrum* and *Halobacterium* showed significantly negative correlation (adjusted *P* < 0.05) with several lipids, i.e. total cholesterol (TotalC) and low-density lipoprotein cholesterol (LDLC). In addition, we observed a positive correlation (adjusted *P* < 0.05) between the genus *Halolamina* and renal functions (estimated glomerular filtration rate using the MDRD formula, eGFR_MDRD and estimated glomerular filtration rate using the CKD-EPI formula, eGFR_CKDEPI; Fig. 6, right). We also found several positive/negative correlations of the haloarchaeal taxa with food categories (see Additional file 1: Supplementary Fig. S5). However, these results implied no common (dietary) factor influencing the abundance of haloarchaea in the human gut.

### Comparison of the human gut archaeome with the gut archaeomes of the great apes

We observed the assortative characteristics of the four distinct enterotypes (Fig. 3d). As the host diet might affect the formation of enterotypes [29], the archaeal enterotypes may have arisen over relatively recent timescales in the human lineage. However, Fig. 6 and Additional file 1: Supplementary Fig. S5 show weak (or no) relationship between archaeal abundances and host dietary factors. Next, we assessed whether these enterotypes are the results of more ancient features (such as host immune system and gut physiology) that might be derived before or during the diversification of the great ape species by comparing the Korean gut archaeome with other human gut archaeomes [11, 28] and those of the great apes, including orangutan, gorilla, chimpanzee and bonobo [30]. PCoA of the Bray-Curtis dissimilarity matrix showed the typical horseshoe shape of all merged samples (see Additional file 1: Supplementary Fig. S6a), indicating high dissimilarity among the gut archaeomes of the great apes. Comparison of the human and non-human samples revealed that weighted PCoA (see Additional file 1: Supplementary Fig. S6a) and unweighted PCoA (based on the Jaccard dissimilarity matrix; see Additional file 1: Supplementary Fig. S6b) showed significantly separated community structure and composition of the gut archaeome, respectively (permutational multivariate analysis of variance [PERMANOVA], *P* = 0.001, comparison between the human and non-human samples). UPGMA clustering analysis showed that the community structure of the gut archaeome of the great apes (except those of humans) appeared to mimic the host phylogeny, as shown by the absence of any significant difference in the distance between chimpanzee and bonobo gut archaeome (see Additional file 1: Supplementary Fig. S6c). Neither the community structure nor the composition of the human gut archaeome was closely related to those of chimpanzees or bonobos (see Additional file 1: Supplementary Fig. S6c and d), suggesting that the human gut archaeome is distinct and not related to host phylogeny.

## Discussion

Here, we reported the human gut archaeome of 897 Korean subjects. Using archaeal metataxonomic analysis with nested PCR amplicons and an archaea-specific primer set, we were able to detect archaeal sequences from 381 samples (42.47%, Fig. 1). This distribution ratio of the archaeal 16S rRNA gene sequence-positive samples is in accordance with the results of a previous study showing archaeal colonisation in the gastrointestinal tract of approximately half of the human population [31]. Furthermore, we observed the abundance of the human gut archaea (10.24 ± 4.58% of the total bacterial and archaeal abundance; Fig. 2). Archaeal abundance in the human gut has been estimated previously, ranging from 0.1 to 21.3% [11, 32]. Our abundance estimation did not vary substantially from those of previous studies, although the values were based on a relatively larger sample size (*n* = 150), thereby enhancing the reliability of the estimation. Both the prevalence and the quantitative data obtained collectively supported the robustness of our methodological approach, which minimised the experimental pitfalls in human archaeome analysis. Given that the adjusted value of the gut archaeal abundance based on the archaeal/bacterial 16S rRNA gene copy number per genome showed over a twofold increase in estimated abundance of the human gut archaea (Fig. 2), the quantitative characteristics of the gut archaeome and their net effect(s) on host physiology remain to be elucidated.

PCR amplicon sequencing analysis revealed that sequences assigned to the genus *Methanobrevibacter* and *Methanosphaera* are most proportionally abundant in all faecal samples where archaea are detected. These two genera contain strictly anaerobic methanogens utilising hydrogen/carbon dioxide and hydrogen/methanol, respectively [17], and are capable of entering into syntrophic relationships with gut bacterial microbiota by consuming the excess hydrogen produced during anaerobic fermentation of carbohydrates, thereby increasing the ATP synthesis of anaerobic bacteria and promoting the growth of gut bacteria [12]. In contrast, gut methanogens have been correlated with various diseases (i.e. colorectal cancer, obesity, anorexia, inflammatory bowel disease, irritable bowel syndrome, diverticulosis, constipation and periodontitis) in humans [33–35]; but the results are inconsistent, and are dependent on the methodology used. Correlation coefficient analysis on the abundance of methanogens and host clinical phenotypes did not show any relationship between the two variables. We instead observed that the genus *Methanosphaera* was positively related to host dietary nutrients, suggesting that the abundance of some specific members of the human gut archaeome might be orthogonally manipulated by host dietary nutrients.

In particular, this study demonstrated the extensive colonisation of the Korean gut by haloarchaea. We detected haloarchaea-assigned phylotypes in 364 out of 381 archaea-positive faecal samples (95.54%). Haloarchaea were more prevalent than methanogens in some samples. To our knowledge, no metataxonomic study has reported haloarchaea-assigned sequences in the human gut. A recent study by Oxley et al. reported the possible presence of haloarchaea in the human intestinal mucosa, although haloarchaea has not yet been confirmed as indigenous gut microbiota because (i) the results were based on an insufficient number of sequences obtained from the clone libraries, and (ii) the study was biased toward the diseased samples (i.e. they obtained mucosal biopsies from the colons of patients with inflammatory bowel disease) [36]. Using a culture-dependent approach, Seck et al. recently isolated two haloarchaeal strains from 572 human faecal samples. They subsequently conducted metataxonomic analysis using the archaeal 16S rRNA gene amplicon sequencing of 164 human faecal samples but did not detect haloarchaea-assigned sequences [19].

Haloarchaea includes salt-loving microorganisms, which have been considered extremophiles as they were frequently detected in hypersaline environments. The genus *Haloplanus* consists of eight species isolated from solar salterns, aquaculture farms, crude solar salts, and Dead Sea–Red Sea water mixtures [37]. They can grow in medium containing 0.9–5.1 M NaCl, and some species grow anaerobically in the presence of nitrate, dimethylsulphoxide (DMSO) or l-arginine. The genus *Halorubrum* consists of 37 species isolated from hypersaline environments, such as rock salt and solar saltern [38]. They grow in medium with 1.0–5.2 M NaCl and some species grow under anaerobic conditions in the presence of nitrate, DMSO or l-arginine. Various haloarchaeal strains were also isolated from food products and ingredients of salt-fermented food products [39]. In Korea, salt is widely used in fermented foods as the oldest food preservation technique for inhibiting the growth of unfavourable bacteria. Hence, we attempted to identify the factor(s) affecting the high prevalence/abundance of gut haloarchaea from dietary habits; however, no positive relationship was observed between the relative abundance of haloarchaea and host dietary nutrients/salt-containing food items (Fig. 6 and Supplementary Fig. S5). This was not surprising as fermented food products are daily consumed by billions of people worldwide [40]. Therefore, investigations regarding the mechanisms underlying haloarchaeal dissemination in the human gut are essential.

We next investigated if the gut haloarchaea are members of the human natural gut microbiota or if they reflect foodborne microbiota that transiently passes through the gut. From the metataxonomic and FISH analyses, we could not confirm whether the observed haloarchaea are colonisers of the gut, merely passing through after environmental exposure, or were consumed in the diet. Given that Korean guts possessed a structured community of haloarchaea with a complex composition of haloarchaeal phylotypes (Figs. 3 and 4), the possible growth and development of haloarchaea in the human gut should be investigated further. As shown in Additional file 1: Supplementary Fig. S4, the mean faecal salinity in Korean subjects was 0.68%, ranging from 0.30 to 1.05%. Haloarchaea is increasingly detected in habitats of relatively low salinities [41, 42], supporting the possible growth of haloarchaea in the human gut. Higher faecal salinity might not necessarily promote haloarchaeal growth in the human gut, as evidenced by the absence of haloarchaea-assigned sequence, in which the mean salinity is 0.7% and ranging from 0 to 6% [19]. Members of haloarchaea can grow either aerobically or anaerobically. The nutritional demands and metabolic pathways for aerobic heterotrophic metabolism of haloarchaea are diverse [43]. However, the normal human gut is characterised by anaerobic conditions with extremely low quantity of oxygen in the luminal space maintained by the oxygen consumption of the host cells [44, 45]. Oxygen has low solubility in salt-saturated natural environments and is possibly a limiting resource for haloarchaea growth. Under these conditions, many haloarchaeal members grow as facultative anaerobic heterotrophs using alternative electron acceptors (such as nitrate, trimethylamine *N*-oxide, fumarate, thiosulphate or elemental sulphur) [43] for metabolism and fermentation of l-arginine, as well as using strictly anaerobic acetotrophic metabolism with acetate as the electron donor and elemental sulphur as the electron acceptor [46], and strictly anaerobic lithoheterotrophic metabolism with hydrogen as the electron donor and elemental sulphur or thiosulphate as electron acceptor [47]. A culture-dependent approach, along with confirmation of the existence of the haloarchaeal phylotypes based on culture-independent methods, combined with the use of a gnotobiotic animal model (i.e. animals harbouring a defined haloarchaeal communities in their gut) will further elucidate the growth and development of anaerobic heterotrophic or undiscovered ecotypic haloarchaea in the gut environment.

We also categorised the Korean gut archaeome into four archaeal enterotypes: the *Methanobacteriaceae*-, *Methanomethylophilaceae*-, *Haloferacaceae*- and the unclassified *Euryarchaeota*-dominant archaeome (Fig. 3). These enterotypes reflect the proportional categories (or patterns) within the gut microbiota in different individuals, thereby being the well-balanced and quite stable microbial members among all individuals [27]. However, a careful interpretation is needed as the categorisation of the gut microbiota based on the dominance of certain genera may result in oversimplification [48]. Further studies to clarify the archaeal complexity within the gut and to link it to clinical traits will be of interest.

## Conclusions

We conducted a population-level metataxonomic analysis on the gut archaeome of Koreans. Our deep sequencing analysis revealed unexpectedly diverse archaeal communities in the human gut. Interestingly, we observed extensive colonisation of haloarchaea in the Korean gut. However, our study has some limitations; we could not show that the abundant archaeal species belonging to both the methanogens and haloarchaea (identified from metataxonomic analysis) consisted of members of the viable natural gut microbiota. Further identification of the spatiotemporal dynamics of the human gut archaeome using longitudinal samples from different compartments of the intestinal tract should be performed. In addition, the clustering results of the archaeal communities in human and non-human samples (shown in Additional file 1: Supplementary Fig. S6) might be biased due to methodological differences used in each study. Nonetheless, we observed (i) an unexpectedly diverse archaeal community in the human gut and (ii) inter-individual variation in the human gut archaeome characterised by the presence of several clusters of gut archaeal enterotypes. Collectively, our results have expanded our understanding of the human gut archaeome, suggesting that the human gut archaeome is indigenous, responsive, and functional.

## Methods

### Sample preparation and DNA extraction

A total of 897 subjects were enrolled in the Korean gut microbiome project. Subjects who had consumed antibiotics in the 3 months before the study or those with a history of major gastrointestinal diseases were excluded. Subjects consisted of 203 males and 694 females, and their age ranged from 20 to 90. All subjects completed a questionnaire that covers comprehensive demographic and lifestyle information and a food frequency questionnaire (FFQ) [49]. Subjects underwent blood biochemical tests and anthropometrical measurements. Metadata, including clinical measurements and food frequency details, are available in the independent study by Lim et al*.* [50]. Participants’ faecal samples were collected in both an OMNIgene-GUT tube (DNA Genotek, Ontario, Canada) and a sterile stool collection container. Microbial DNA was extracted using the QIAamp DNA stool mini kit (QIAGEN, Hilden, Germany) with additional bead-beating and heating steps [51]. The elution buffer volumes were 200 μL, and the extracted DNA samples were stored at − 20 °C until further use.

### Library preparation and sequencing of archaeal 16S rRNA gene

To amplify the archaeal 16S rRNA genes, a nested PCR was performed as described previously [28] using MG 2X PCR mastermix (MGmed, Seoul, South Korea) and Accupower™ Hotstart PCR premix (Bioneer, Daejeon, South Korea) in the first (25 cycles) and second PCRs (25 cycles), respectively. Briefly, we used the primer pairs S-D-Arch-0344-a-S-20 and S-D-Arch-0911-a-A-20 in the first PCR, and S-D-Arch-0349-a-S-17 and S-D-Arch-0519-a-A-16, which included the Illumina adapter sequence, in the second PCR. The primer sequences are listed in Additional file 2: Supplementary Table S3. Three PCR products with the same template were pooled. To purify the products obtained in the first and second rounds, x-tracta™ Gel Extractor (Promega, Madison, WI, USA) and Qiaquick PCR & gel cleanup kit (QIAGEN) were used. Among the 897 samples, 381 were confirmed using electrophoresis on 1% agarose gel. To verify the size of the PCR-enriched fragments, template size distribution was checked using the Agilent Technologies 2100 bioanalyzer and a DNA 1000 chip. Libraries for archaeal 16S rRNA gene sequences were constructed using the Nextera DNA Flex library preparation kit (Illumina, San Diego, CA, USA), following the manufacturer’s instruction. A subsequent limited-cycle amplification step was performed to add multiplexing indices and Illumina sequencing adapters. The final products were normalised and pooled using PicoGreen, and the size of the libraries was verified using the TapeStation DNA screentape D1000 (Agilent, Santa Clara, CA, USA). Sequencing was performed using the HiSeq™ X platform (Illumina). We also investigated possible DNA contamination in all reagents used for DNA extraction. PCR analysis targeting the archaeal 16S rRNA gene (25 + 25 cycle reactions) revealed no apparent contamination (Additional file 1: Supplementary Fig. S7a). PCR amplicons from ‘blank’ negative DNA extraction/PCR controls (i.e. PCR products of template acquired from a sham extraction to which no faecal sample is added; *n* = 3) were used as the negative controls (Additional file 1: Supplementary Fig. S7b).

### Analysis of 16S rRNA gene sequence data

By using the bcl2fastq2 conversion software v. 2.20.0. (Illumina), the adapter sequences were trimmed from the raw FASTQ reads, and the trimmed reads were demultiplexed according to the samples. To generate the ASVs feature table, the sorted reads were imported and processed using QIIME2 v. 2018.11 [52]. In total, 275,909,328 imported paired reads were quality filtered, denoised and merged using the DADA2 plugin [53]. Chimeric sequences and singleton ASVs were excluded in further analyses. Rarefaction curves were constructed using plugin diversity alpha-rarefaction in QIIME2. For taxonomic classification, we used plugin q2-feature-classifier, the classify-sklearn method [54] and the pre-trained SILVA v. 132 database [55] with 99% identity. An overview of the human gut archaeal 16S rRNA gene sequence dataset is provided in Additional file 2: Supplementary Table S4.

To calculate the 16S rRNA gene similarities with the secondary structure, the ASVs were aligned using the RDP aligner (https://pyro.cme.msu.edu/aligner/form.spr). Afterwards, the aligned ASVs were used to construct a phylogenetic tree using the neighbour joining algorithm based on the Kimura 2-parameter model with 1000 bootstraps in MEGA X [56]. Phylogenetic tree visualisation was performed using iTOL v. 5 [57]. Phylogenetic analysis of the haloarchaea-assigned sequences included 16S rRNA gene sequences of the type strains belonging to the class *Halobacteria* and clone sequences from Oxley et al. [36], whereas that of the methanogen-assigned sequences included 16S rRNA gene sequences of the type strains belonging to the genera *Methanobrevibacter* and *Methanosphaera* and the family *Methanomethylophilaceae*. To determine the species diversity in each human faecal sample, alpha and beta diversity analyses were performed using the plugin q2-diversity in QIIME2 v. 2018.11. Based on rarefaction results, we subsampled the sequences at a sampling depth of 10,000 (Additional file 1: Supplementary Fig. S1) and included 342 from the 381 archaeal sequence-positive samples. Subsequently, group difference was determined based on metadata.

Archaeal enterotypes were identified as previously described [58]. Briefly, the samples were clustered using PAM clustering [59] with the Bray-Curtis dissimilarity matrix [60] at the family level. The optimal number of clusters was determined using the silhouette index [61]. Enterotypes were visualised by the PCoA plot. To find the sequences assigned to haloarchaea in other sample cohorts, we trawled both the publicly available human metagenomic and metataxonomic datasets using the EBI MGnify [62]. All the data were obtained from the EBI MGnify database.

The archaeal 16S rRNA dataset generated using HiSeq for the negative controls is summarised in Additional file 2: Supplementary Table S5. The taxonomic annotation data for the negative controls are shown in Additional file 2: Supplementary Table S6. Majority of the assigned reads in the negative controls were highly unlikely to be present in the human gut, suggesting no (or very little) impact of contamination on the archaeal 16S rRNA gene analysis.

### Estimation of archaeal abundance

DNA was extracted from the faecal samples as described above. In total, 150 samples were randomly selected and subjected to real-time quantitative PCR [11] in three replicates. The archaeal primer sequences are listed in Additional file 2: Supplementary Table S3. Bacterial 16S rRNA gene (primers Bac1055YF and Bac1392R) was used as the control [63]. PCR was performed using the CFX96™ real-time PCR detection system (Bio-Rad, Hercules, CA, USA) in a reaction volume of 20 μL containing 10 μL TOPreal qPCR 2X premix (Enzynomics, Daejeon, South Korea), 300 nM of each of the forward and reverse primers and 2 ng template DNA. The Cq values were determined using the Bio-Rad CFX Manager software version 3.1. *Escherichia coli* K12 and *Haloplanus salinus* JCM 18368^T^ were used to construct standard curves and perform quantitative analysis. The PCR efficiency and *R*^2^ were 96.92% and 0.9995 for bacteria, and 97.57% and 0.9983 for archaea, respectively.

### Fluorescence in situ hybridisation analysis

Fluorescence in situ hybridisation (FISH) analysis was performed according to a method by Hugenholtz [64] with minor modification. Briefly, the haloarchaea-specific probe (HALO775) was designed using the ARB software [65]. The specificity of the HALO775 was evaluated based on the TestProbe in SILVA (SILVA SSU database v. 138) and the ProbeMatch in ribosomal database project (RDP, v. 11.5) databases (Additional file 2: Supplementary Tables S1 and S2). The designed probe, the bacterial 16S rRNA gene-targeting probe EUB338 [66] and non-specific probe NONEUB [67] were synthesised and labelled at the 5′ end with Cy3, Cy5 and FAM by Macrogen (Seoul, South Korea), respectively. To evaluate the possible cross-binding activity, we tested the binding activities of HALO775 to a haloarchaeon (*Haloplanus salinus* JCM 18368^T^), bacterium (*Escherichia coli* K12) and another archaeon (*Methanobrevibacter smithii* JCM 30028^T^). The cultured bacterium and the archaea were fixed with 4% paraformaldehyde in PBS at 4 °C for 4 h, and the fixed samples were washed in PBS and gradually dehydrated in PBS-ethanol solution (final ratio of 1:1, vol/vol). Hybridisation was performed at 46 °C with 20% formamide hybridisation buffer. Afterwards, washing was performed again at 48 °C. Both non-binding probe (NONEUB_FAM_) and no-probe controls were always included to decipher false positives. Samples were observed under a confocal microscope (LSM710; Carl Zeiss, Oberkochen, Germany) with × 1000 magnification using the imaging software of ZEN v. 3.1 (blue edition, Carl Zeiss).

### Measurements of salinity and inorganic elements

According to the relative abundance of haloarchaea, 20 faecal samples were selected. Each specimen (200 mg) was diluted in 1 mL distilled water, and the salinity was measured using a salinity refractometer (Atago, Japan). The inorganic elements from these samples were measured using ICP-MS, as previously described [68].

### Statistical analysis

Normality tests (Shapiro-Wilk) were carried out prior to correlation coefficient analysis and comparison of multiple samples. Correlation coefficient analysis was performed based on the relative abundance of the proportionally abundant archaeal taxa with respect to dietary nutrients, clinical metadata and food categories. Multiple samples were compared using the nonparametric Kruskal-Wallis test, followed by Dunn’s multiple comparisons test. PERMANOVA analysis was done based on the Bray-Curtis and Jaccard dissimilarity matrices, with 999 permutations. All statistical analyses were performed using the GraphPad Prism software v. 7.05 (*P < 0.05, **P < 0.01 and ***P < 0.001).

## Supplementary information

**Additional file 1.** Supplementary Figures (S1-S7).

**Additional file 2.** Supplementary Tables (S1-S6).

## Data Availability

The sequencing reads have been deposited in the NCBI under the accession number PRJNA522626.

## Abbreviations

ASV: Amplicon sequence variant
DMSO: Dimethylsulphoxide
FISH: Fluorescence in situ hybridisation
ICP-MS: Inductively coupled plasma-mass spectrometer
LDLC: Low-density lipoprotein cholesterol
PAM: Partitioning around medoids
PCoA: Principal coordinate analysis
PCR: Polymerase chain reaction
TotalC: Total cholesterol
