# Supplementary material for: The human gut archaeome: identification of diverse haloarchaea in Korean subjects
Source: Microbiome. 2020 Aug 4;8:114. doi: 10.1186/s40168-020-00894-x (PMC7409454; doi:10.1186/s40168-020-00894-x)
Supplement: Supplementary file 2 — Additional file 1. Supplementary Figures (S1-S7). [file 40168_2020_894_MOESM1_ESM.docx]

Supplementary Figures for

**The human gut archaeome: identification of diverse haloarchaea in Korean subjects**

Joon Yong Kim^1^, Tae Woong Whon^1^, Mi Young Lim^2^, Yeon Bee Kim^1^, Namhee Kim^1^, Min-Sung Kwon^1^, Juseok Kim^1^, Se Hee Lee^1^, Hak-Jong Choi^1^, In-Hyun Nam^3^, Won-Hyong Chung^2^, Jung-Ha Kim^4^, Jin-Woo Bae^5^, Seong Woon Roh^1,^*, Young-Do Nam^2,^*

^1^Microbiology and Functionality Research Group, World Institute of Kimchi, Gwangju 61755, Republic of Korea

^2^Research Group of Healthcare, Research Division of Food Functionality, Korea Food Research Institute, Jeollabuk-do 55365, Republic of Korea

^3^Geologic Environment Division, Korea Institute of Geoscience and Mineral Resources, Daejeon 34132, Republic of Korea

^4^Department of Family Medicine, Chung-Ang University Hospital, Chung-Ang University College of Medicine, Seoul 06973, Republic of Korea

^5^Department of Biology, Kyung Hee University, Seoul 02447, Republic of Korea

*Correspondence: swroh@wikim.re.kr (S.W.R.), youngdo98@kfri.re.kr (Y.-D.N.)

Supplementary Figures S1–S7


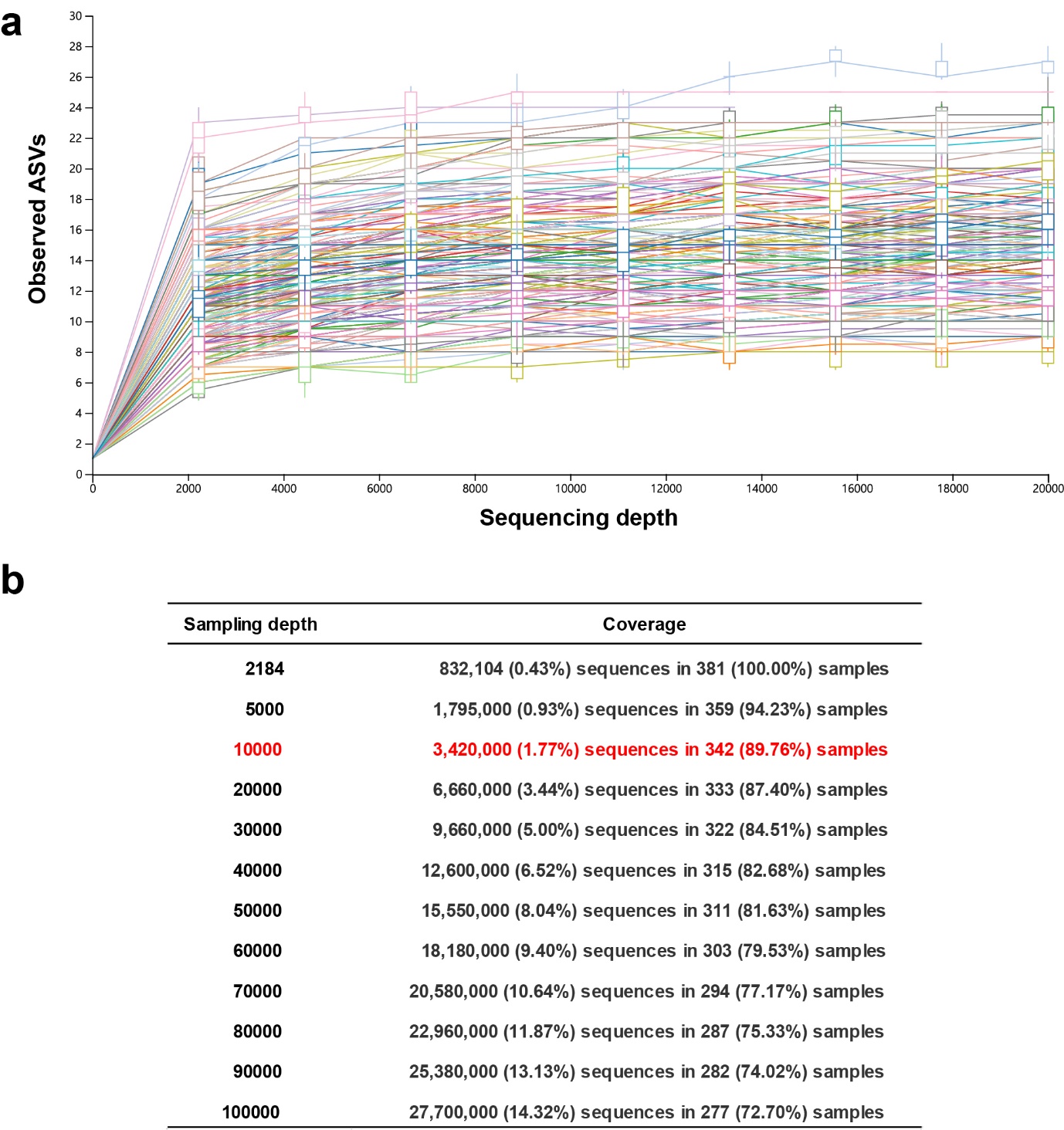


**Supplementary Fig. S1.** Sequencing depth of the human gut archaeal 16S rRNA gene sequence dataset. **a**. Rarefaction curves showing the sequencing numbers and observed amplicon sequence variants (ASVs) obtained by QIIME2. Each coloured line represents a sample. **b**. Coverage of all the samples in response to different subsampling depths.


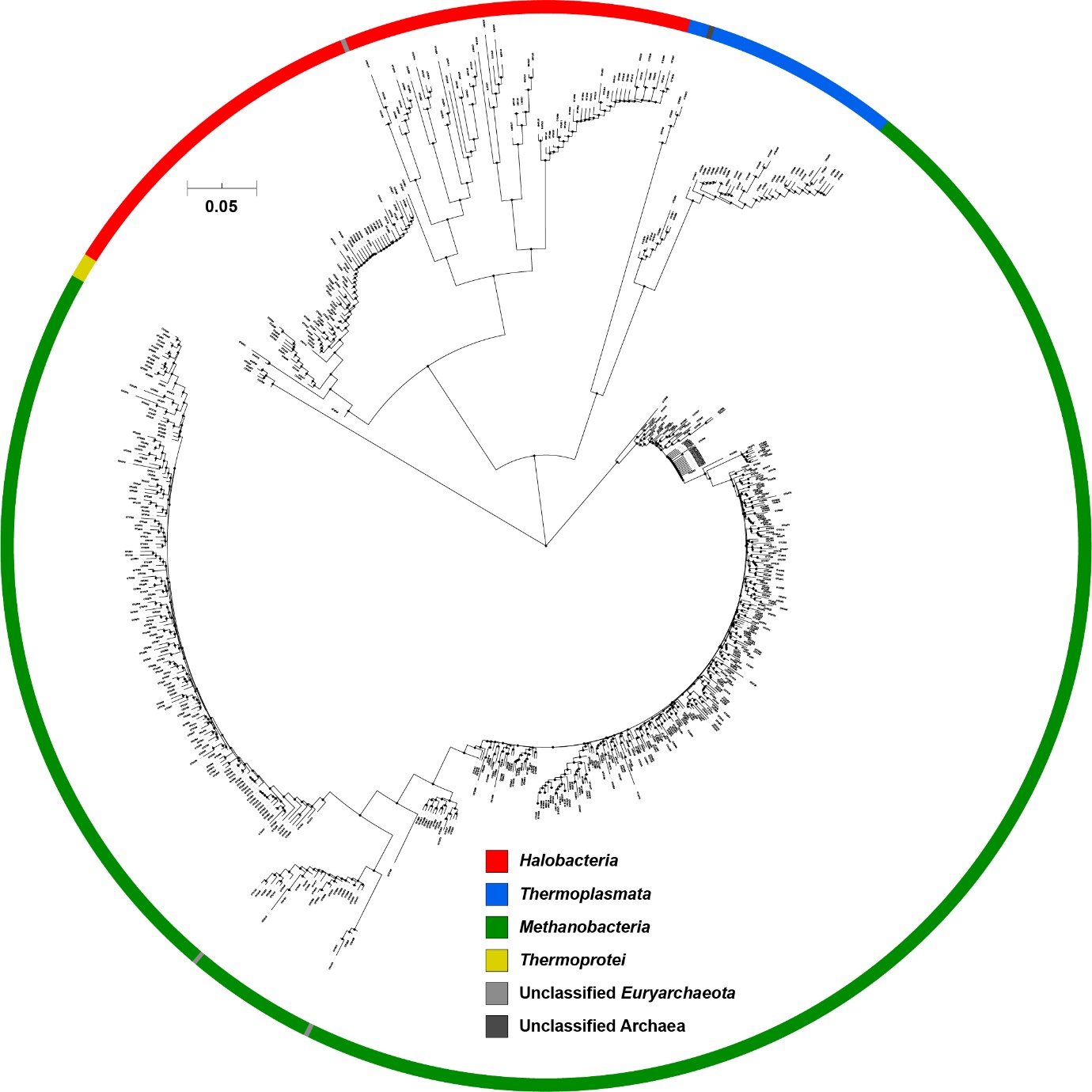


**Supplementary Fig. S2.** Phylogenetic analysis of the total amplicon sequence variants (ASVs). A phylogenetic consensus tree based on the 16S rRNA gene sequences was reconstructed using the neighbor-joining algorithm, indicating the taxonomic positions of total ASVs. Bar corresponds to 0.05 accumulated changes per nucleotide.


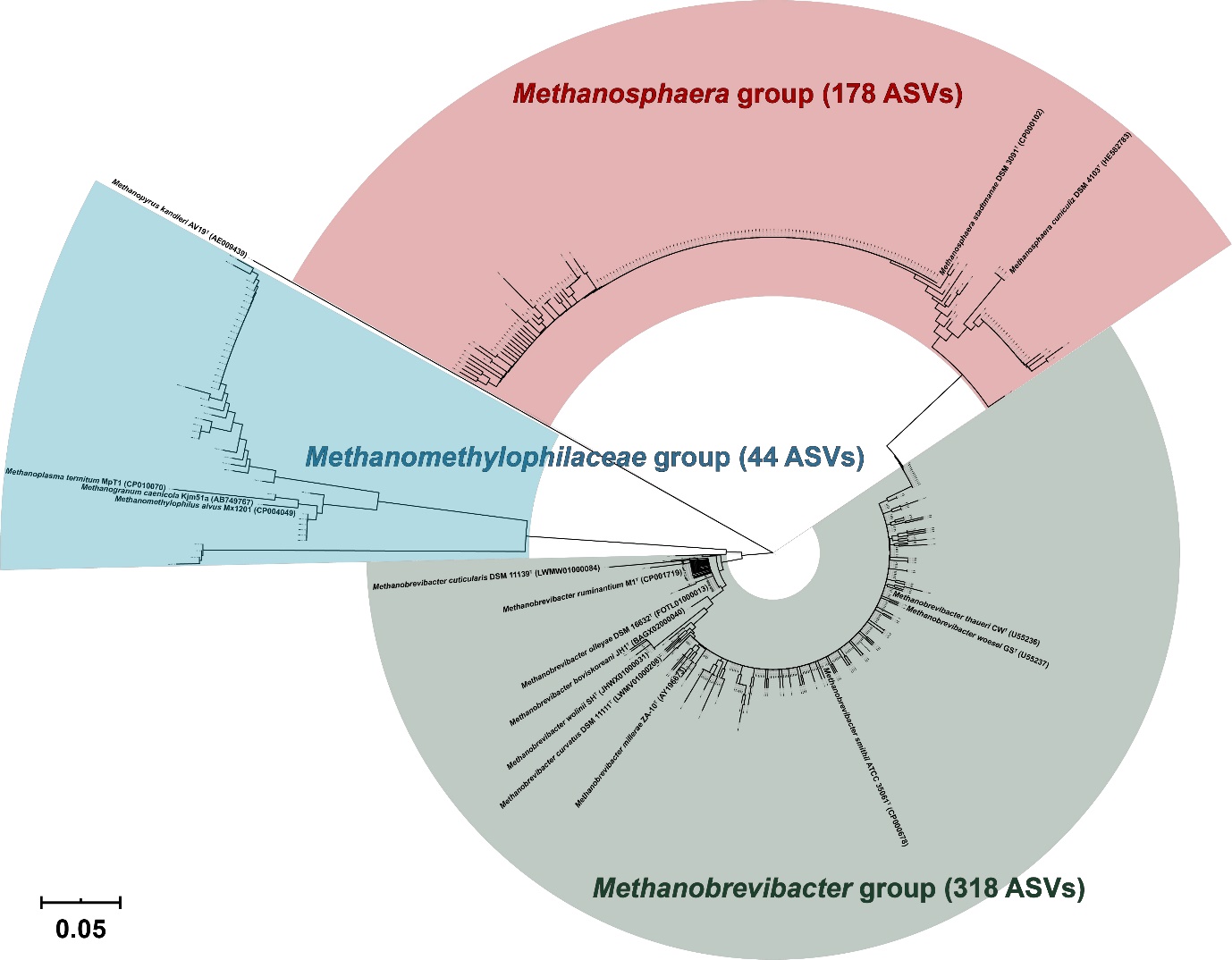


**Supplementary Fig. S3.** Phylogenetic analysis of amplicon sequence variants (ASVs) assigned to methanogens. The ASVs were identified in the faecal archaeal 16S rRNA gene sequence data of the 381 healthy Koreans. The 16S rRNA gene sequences of the validated methanogen species were included. A phylogenetic consensus tree based on the 16S rRNA gene sequences was reconstructed using the neighbor-joining algorithm, indicating the taxonomic positions of the ASVs assigned to the methanogens. Bar corresponds to 0.05 accumulated changes per nucleotide.


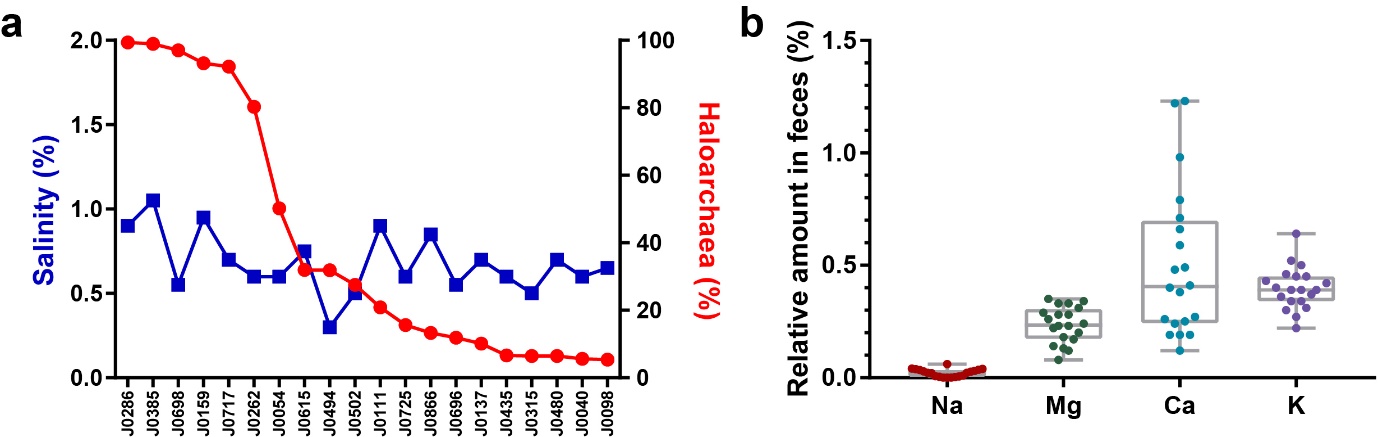


**Supplementary Fig. S4.** Measurements of salinity and inorganic elements. Twenty faecal samples were randomly selected containing the haloarchaea-assigned sequences. **a**. Faecal salinity was measured using a salinity refractometer. The proportional abundances of haloarchaea (relative to total archaea) were obtained from the metataxonomic analysis. **b**. Relative amounts (weight per gram of faeces) of the salt-consisting inorganic elements were measured using an inductively coupled plasma-mass spectrometer (ICP-MS). Total faecal weight was taken arbitrarily as 100. The lines, boxes, and whiskers in the box plot diagrams represent the median, first, and third quartiles, and min-to-max distribution of replicate values, respectively. The values and scattered dots in the bar graphs represent the means ± SEM and the individual replicates, respectively.


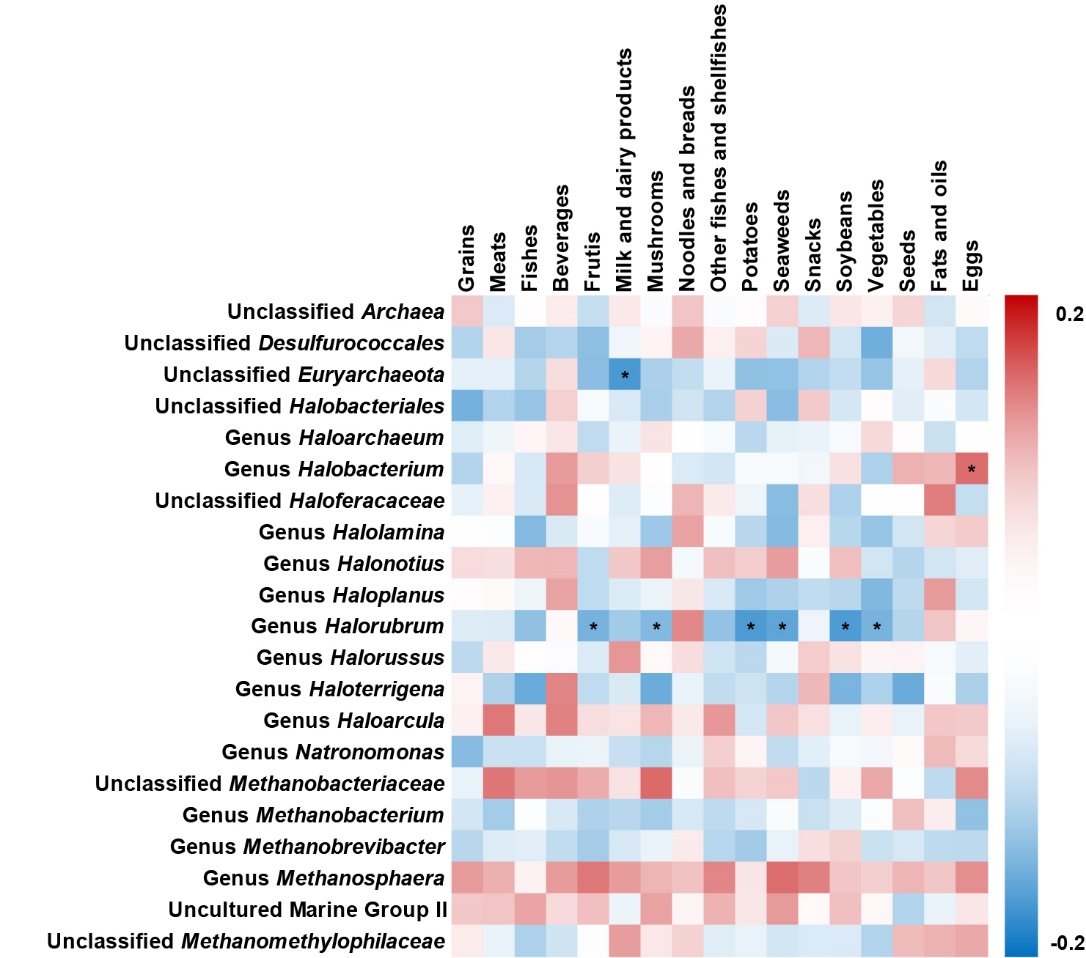


**Supplementary Fig. S5.** Correlation analysis of archaeal taxa and food types (host factor). Spearman’s rank correlation coefficients and the corresponding *P*-values were calculated based on comparisons of the relative abundance of the abundant archaeal taxa at the genus level and ingested food types. For orphan sequences (i.e., unclassified at the genus level), a high-rank lineage is provided. A correction for multiple comparisons was based on the false discovery rate (FDR, threshold of 0.05). *Adjusted *P* < 0.05.


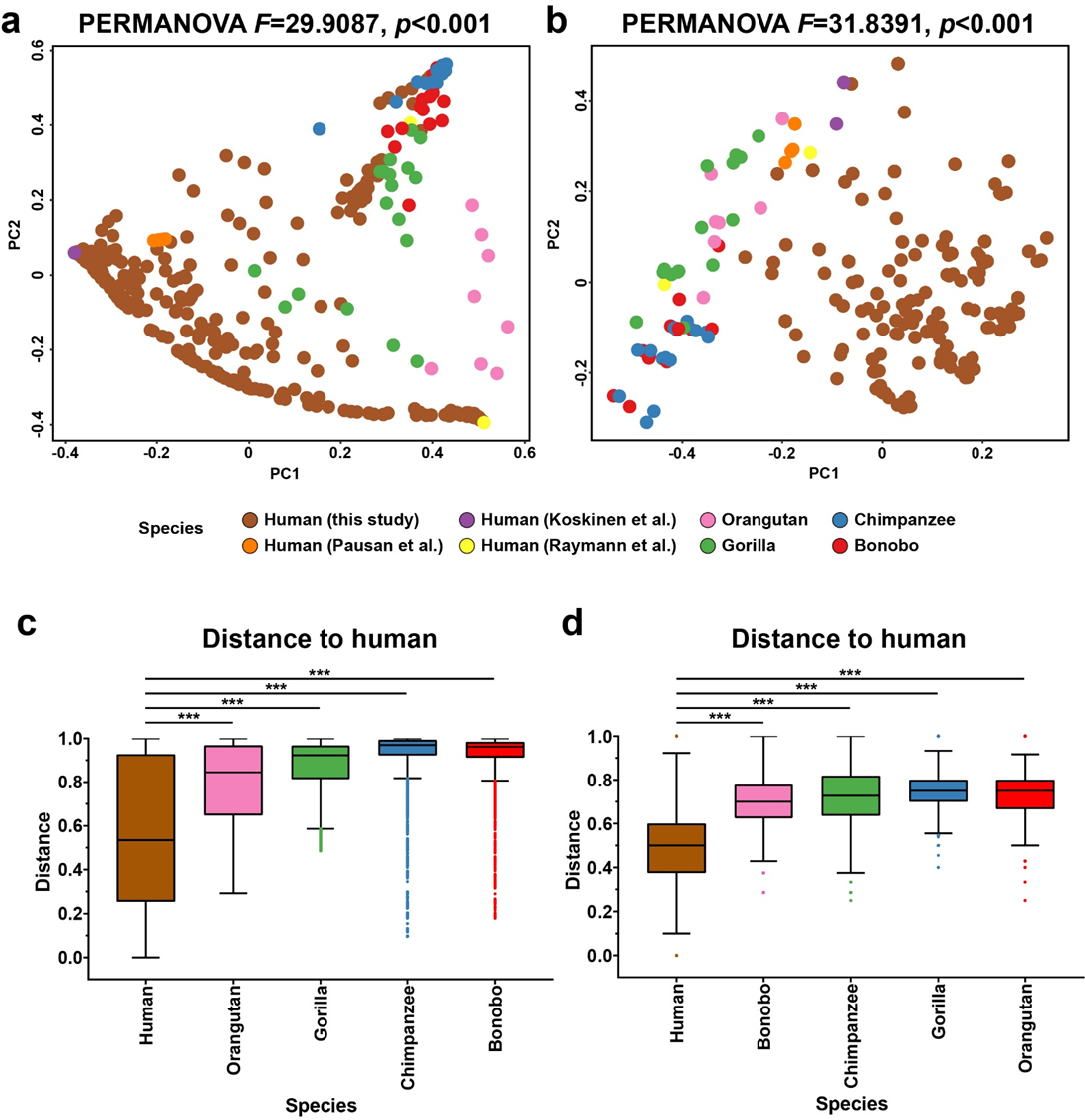


**Supplementary Fig. S6.** Comparative analysis of gut archaeomes of human and great apes. The human gut archaeal 16S rRNA gene sequences were collected from Koreans (*n* = 342, from the 381 archaeal sequence positive samples at a sampling depth of 10,000), Europeans (Pausan et al. [1], *n* = 5; Koskinen et al. [2], *n* = 6), and Americans (Raymann et al. [3], *n* = 2). For the non-human samples, we included the gut archaeal 16S rRNA gene sequences of orangutans (*Pongo pygmaeus*, Atlanta Zoo in USA, *n* = 8), gorillas (*Gorilla gorilla*, Cameroon, *n* = 18), chimpanzees (*Pan troglodytes*, Tanzania, *n* = 14), and bonobos (*Pan paniscus*, DR Congo, *n* = 17) [3]. **a**. Weighted PCoA was generated based on the Bray-Curtis dissimilarity matrix. **b**. Unweighted PCoA was generated based on the Jaccard dissimilarity matrix. **c, d**. Distances between groups were calculated based on the Bray-Curtis dissimilarity matrix (c), and the Jaccard dissimilarity matrix (d). The lines, boxes, and whiskers in the box plot diagrams represent the median, first, and third quartiles, and min-to-max distribution of replicate values, respectively. The values and scattered dots in the bar graphs represent the means ± SEM and the individual replicates, respectively. The data were analysed using PERMANOVA with 999 permutations (comparison between human and non-human; a and b), and the nonparametric Kruskal-Wallis test, followed by Dunn’s multiple comparisons test (c and d). ****p* < 0.001, ns: not significant.


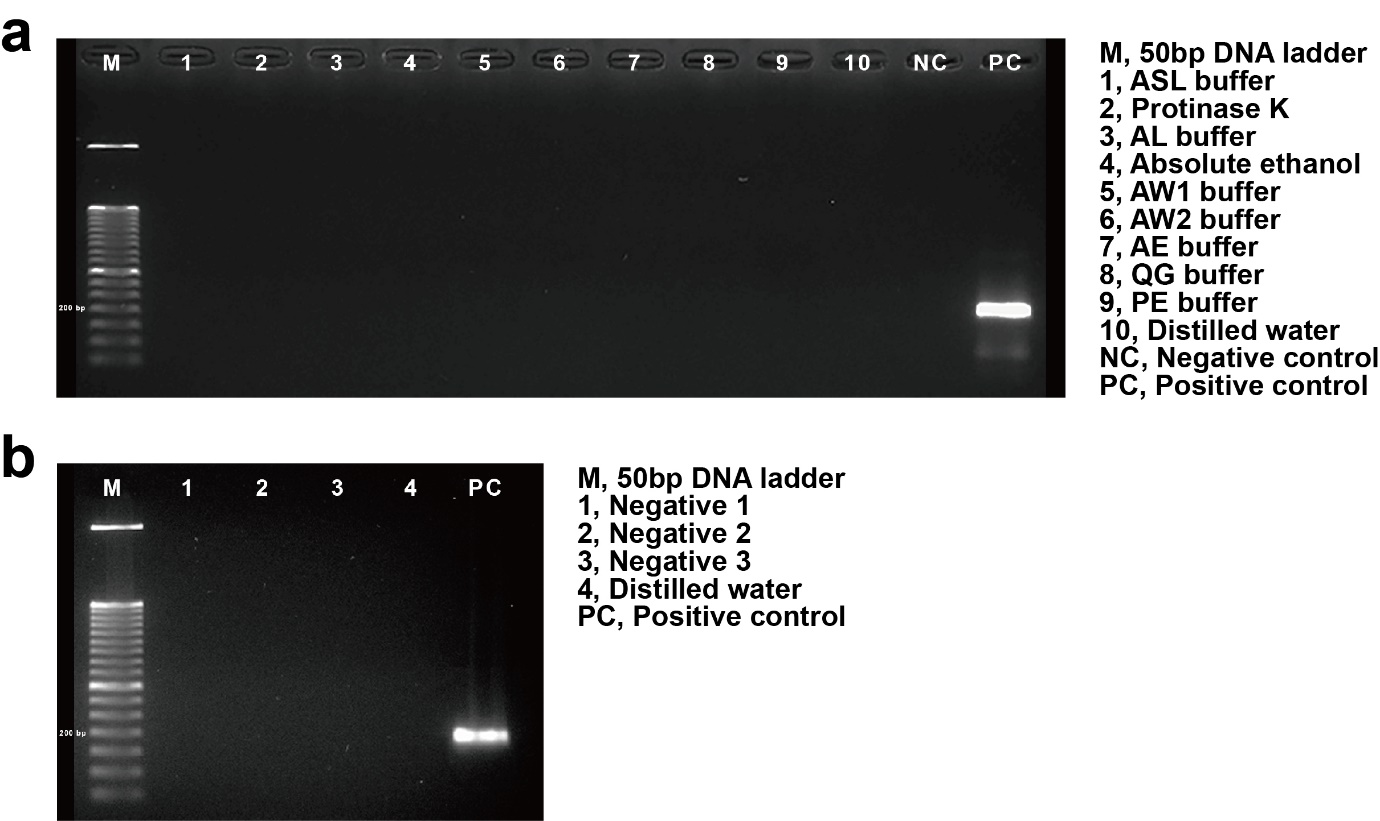


**Supplementary Fig. S7.** Monitoring DNA contamination for the archaeal 16S rRNA gene sequence analysis. **a**. The possible DNA contamination of all the reagents used for DNA extraction was tested by PCR analysis, targeting the archaeal 16S rRNA gene (25 + 25 cycle reactions). **b**. A band image for the "blank" negative DNA extraction/PCR controls (i.e., PCR products of template acquired from a sham extraction to which no faecal sample is added) with three biological replicates.

**References**

1. Pausan MR, Csorba C, Singer G, Till H, Schopf V, Santigli E, Klug B, Hogenauer C, Blohs M, Moissl-Eichinger C. Exploring the Archaeome: Detection of Archaeal Signatures in the Human Body**.** Front Microbiol. 2019;10.

2. Koskinen K, Pausan MR, Perras AK, Beck M, Bang C, Mora M, Schilhabel A, Schmitz R, Moissl-Eichinger C. First Insights into the Diverse Human Archaeome: Specific Detection of Archaea in the Gastrointestinal Tract, Lung, and Nose and on Skin**.** mBio. 2017;8**:**e00824-17.

3. Raymann K, Moeller AH, Goodman AL, Ochman H. Unexplored Archaeal Diversity in the Great Ape Gut Microbiome**.** mSphere. 2017;2.
