# Supplementary material for: The human gut archaeome: identification of diverse haloarchaea in Korean subjects
Source: Microbiome. 2020 Aug 4;8:114. doi: 10.1186/s40168-020-00894-x (PMC7409454; doi:10.1186/s40168-020-00894-x)
Supplement: Supplementary file 3 — Additional file 2. Supplementary Tables (S1-S6). [file 40168_2020_894_MOESM2_ESM.docx]

Supplementary Tables for

**The human gut archaeome: identification of diverse haloarchaea in Korean subjects**

Joon Yong Kim^1^, Tae Woong Whon^1^, Mi Young Lim^2^, Yeon Bee Kim^1^, Namhee Kim^1^, Min-Sung Kwon^1^, Juseok Kim^1^, Se Hee Lee^1^, Hak-Jong Choi^1^, In-Hyun Nam^3^, Won-Hyong Chung^2^, Jung-Ha Kim^4^, Jin-Woo Bae^5^, Seong Woon Roh^1,^*, Young-Do Nam^2,^*

^1^Microbiology and Functionality Research Group, World Institute of Kimchi, Gwangju 61755, Republic of Korea

^2^Research Group of Healthcare, Research Division of Food Functionality, Korea Food Research Institute, Jeollabuk-do 55365, Republic of Korea

^3^Geologic Environment Division, Korea Institute of Geoscience and Mineral Resources, Daejeon 34132, Republic of Korea

^4^Department of Family Medicine, Chung-Ang University Hospital, Chung-Ang University College of Medicine, Seoul 06973, Republic of Korea

^5^Department of Biology, Kyung Hee University, Seoul 02447, Republic of Korea

*Correspondence: swroh@wikim.re.kr (S.W.R.), youngdo98@kfri.re.kr (Y.-D.N.)

Supplementary Tables S1–S6

**Supplementary Table S1.** The in silico binding specificity of the haloarchaea-specific probe (HALO775) on the TestProbe in SILVA SSU database v. 138.

| **Taxonomy** | **Matched sequence** | **Total sequence** | **Coverage (%)** |
| --- | --- | --- | --- |
| Domain *Bacteria* | 0 | 381,662 | 0.0 |
| Domain *Archaea* | 1,714 | 20,197 | 8.5 |
| Phylum *Halobacterota* | 1,714 | 5,352 | 32.0 |
| Class *Halobacteria* | 1,714 | 2,624 | 65.3 |
| Order *Halobacteriales* | 1,714 | 2,624 | 65.3 |
| Family *Halobacteriaceae* | 133 | 147 | 90.5 |
| Family *Haloferacaceae* | 858 | 1,634 | 52.5 |
| Family *Halococcaceae* | 60 | 86 | 69.8 |
| Family *Halomicrobiaceae* | 610 | 641 | 95.2 |
| Family *Haloadaptaceae* | 0 | 32 | 0.0 |
| Unclassified J07HX5 | 3 | 3 | 100.0 |
| Unclassified *Halobacteriales* | 50 | 77 | 64.9 |
| Phylum *Aenigmarchaeota* | 0 | 319 | 0.0 |
| Phylum *Altiarchaeota* | 0 | 41 | 0.0 |
| Phylum *Asgardarchaeota* | 0 | 670 | 0.0 |
| Phylum *Crenarchaeota* | 0 | 7,625 | 0.0 |
| Phylum *Euryarchaeota* | 0 | 1,993 | 0.0 |
| Phylum *Hadarchaeota* | 0 | 300 | 0.0 |
| Phylum *Hydrothermarchaeota* | 0 | 212 | 0.0 |
| Phylum *Iainarchaeota* | 0 | 46 | 0.0 |
| Phylum *Korarchaeota* | 0 | 62 | 0.0 |
| Phylum *Micrarchaeota* | 0 | 88 | 0.0 |
| Phylum *Nanoarchaeota* | 0 | 550 | 0.0 |
| Phylum *Nanohaloarchaeota* | 0 | 34 | 0.0 |
| Phylum *Thermoplasmatota* | 0 | 2,896 | 0.0 |
| Unclassified *Archaea* | 0 | 8 | 0.0 |
| Domain *Eukaryota* | 0 | 56,836 | 0.0 |

**Supplementary Table S2.** The in silico binding specificity of the haloarchaea-specific probe (HALO775) on the ProbeMatch in ribosomal database project (RDP, v. 11.5).

| **Taxonomy** | **Matched sequence** | **Total sequence** | **Coverage (%)** |
| --- | --- | --- | --- |
| Domain *Bacteria* | 0 | 1,559,421 | 0.0 |
| Domain *Archaea* | 2,143 | 34,907 | 6.1 |
| Phylum *Euryarchaeota* | 2,143 | 17,444 | 12.3 |
| Class *Halobacteria* | 2,143 | 3,161 | 67.8 |
| Order *Halobacteriales* | 996 | 1,182 | 84.3 |
| Family *Halobacteriaceae* | 996 | 1,182 | 84.3 |
| Order *Haloferacales* | 1,129 | 1,372 | 82.3 |
| Family *Haloferacaceae* | 1,129 | 1,372 | 82.3 |
| Order *Natrialbales* | 0 | 570 | 0.0 |
| Unclassified *Halobacteria* | 18 | 37 | 48.6 |
| Class *Archaeoglobi* | 0 | 96 | 0.0 |
| Class *Methanobacteria* | 0 | 3,594 | 0.0 |
| Class *Methanococci* | 0 | 121 | 0.0 |
| Class *Methanomicrobia* | 0 | 4,000 | 0.0 |
| Class *Methanopyri* | 0 | 5 | 0.0 |
| Class *Thermococci* | 0 | 304 | 0.0 |
| Class *Thermoplasmata* | 0 | 4,679 | 0.0 |
| Unclassified *Euryarchaeota* | 0 | 1,484 | 0.0 |
| Phylum *Crenarchaeota* | 0 | 3,044 | 0.0 |
| Phylum *Korarchaeota* | 0 | 97 | 0.0 |
| Phylum *Nanoarchaeota* | 0 | 141 | 0.0 |
| Phylum *Thaumarchaeota* | 0 | 9,349 | 0.0 |
| Phylum *Nanohaloarchaeota* | 0 | 28 | 0.0 |
| Phylum *Woesearchaeota* | 0 | 216 | 0.0 |
| Phylum *Diapherotrites* | 0 | 72 | 0.0 |
| Phylum *Aenigmarchaeota* | 0 | 12 | 0.0 |
| Unclassified *Archaea* | 0 | 4,503 | 0.0 |
| Domain *Fungi* | 0 | 21,369 | 0.0 |

**Supplementary Table S3.** List of PCR primers and FISH probes used in this study.

| Target | Approach | Primer name | Sequence (5'-3') | Reference |
| --- | --- | --- | --- | --- |
| Archaea | Nested 1° | S-D-Arch-0344-a-S-20 | ACGGGGYGCAGCAGGCGCGA | Raskin et al. [1] |
|  |  | S-D-Arch-0911-a-A-20 | GTGCTCCCCCGCCAATTCCT | Stahl et al. [2] |
|  | Nested 2° /  qPCR | S-D-Arch-0349-a-S-17 | GYGCASCAGKCGMGAAW | Takai et al. [3] |
|  |  | S-D-Arch-0519-a-A-16 | TTACCGCGGCKGCTG | Ovreas et al. [4] |
|  | FISH | HALO775 | CTAATCCGGTTCGAGACC | This study |
| Bacteria | qPCR | Bac1055YF | ATGGYTGTCGTCAGCT | Ritalahti et al. [5] |
|  |  | Bac1392R | ACGGGCGGTGTGTAC |  |
|  | FISH | EUB338 | GCTGCCTCCCGTAGGAGT | Amann et al. [6] |

**Supplementary Table S4.** Overview of the human gut archaeal 16S rRNA gene sequence dataset.

| Sample | Input | Quality filtered | Denoised | Merged | Non-chimeric | Singleton-removed | Archaea-assigned |
| --- | --- | --- | --- | --- | --- | --- | --- |
| J0001 | 291,924 | 98,700 | 98,700 | 97,328 | 96,285 | 96,285 | 91,767 |
| J0002 | 520,762 | 179,958 | 179,958 | 169,034 | 164,454 | 164,454 | 35,125 |
| J0003 | 2,221,401 | 2,113,696 | 2,113,696 | 1,624,894 | 1,506,873 | 1,506,873 | 1,433,151 |
| J0004 | 361,071 | 137,388 | 137,388 | 136,408 | 134,670 | 134,670 | 122,155 |
| J0005 | 374,349 | 257,452 | 257,452 | 252,949 | 249,476 | 249,476 | 245,313 |
| J0006 | 352,298 | 244,348 | 244,348 | 241,785 | 237,668 | 237,668 | 235,207 |
| J0007 | 1,874,169 | 1,561,967 | 1,561,967 | 1,549,251 | 1,504,898 | 1,504,898 | 1,483,402 |
| J0008 | 1,523,987 | 1,411,325 | 1,411,325 | 1,406,978 | 1,357,958 | 1,357,958 | 1,355,610 |
| J0009 | 188,186 | 26,218 | 26,218 | 25,641 | 25,446 | 25,446 | 22,503 |
| J0010 | 243,831 | 69,798 | 69,798 | 67,296 | 67,100 | 67,100 | 64,562 |
| J0011 | 277,668 | 142,688 | 142,688 | 142,404 | 141,596 | 141,596 | 140,721 |
| J0012 | 310,071 | 168,277 | 168,277 | 167,288 | 166,074 | 166,074 | 160,729 |
| J0013 | 323,007 | 226,947 | 226,947 | 223,380 | 221,432 | 221,432 | 216,838 |
| J0014 | 361,566 | 218,274 | 218,274 | 208,323 | 207,277 | 207,277 | 201,080 |
| J0015 | 303,245 | 185,485 | 185,485 | 183,786 | 183,041 | 183,041 | 176,695 |
| J0016 | 284,243 | 53,078 | 53,078 | 49,526 | 48,724 | 48,724 | 14,280 |
| J0019 | 1,727,142 | 1,493,628 | 1,493,628 | 1,478,417 | 1,406,816 | 1,406,816 | 1,406,233 |
| J0020 | 1,769,030 | 1,632,302 | 1,632,302 | 1,618,090 | 1,502,588 | 1,502,588 | 1,496,708 |
| J0021 | 319,789 | 236,365 | 236,365 | 234,413 | 232,200 | 232,200 | 230,188 |
| J0022 | 296,937 | 230,817 | 230,817 | 229,616 | 229,490 | 229,490 | 228,644 |
| J0023 | 315,102 | 175,475 | 175,475 | 170,890 | 170,289 | 170,289 | 167,461 |
| J0024 | 1,328,316 | 1,101,459 | 1,101,459 | 1,098,869 | 1,091,280 | 1,091,280 | 1,090,675 |
| J0025 | 1,624,664 | 1,487,304 | 1,487,304 | 1,483,592 | 1,463,650 | 1,463,650 | 1,455,419 |
| J0026 | 300,765 | 235,058 | 235,058 | 231,971 | 227,331 | 227,331 | 225,815 |
| J0027 | 274,402 | 163,377 | 163,377 | 157,173 | 153,488 | 153,488 | 100,383 |
| J0028 | 358,004 | 162,448 | 162,448 | 161,287 | 160,302 | 160,302 | 153,269 |
| J0030 | 286,459 | 139,607 | 139,607 | 139,320 | 138,742 | 138,742 | 136,341 |
| J0031 | 292,150 | 165,553 | 165,553 | 163,569 | 162,154 | 162,154 | 158,995 |
| J0032 | 1,464,777 | 1,259,131 | 1,259,131 | 1,254,956 | 1,211,888 | 1,211,888 | 1,210,812 |
| J0033 | 293,678 | 171,065 | 171,065 | 169,544 | 168,370 | 168,370 | 167,540 |
| J0034 | 344,115 | 211,289 | 211,289 | 210,509 | 173,831 | 173,831 | 171,291 |
| J0038 | 287,147 | 157,205 | 157,205 | 156,618 | 156,557 | 156,557 | 155,860 |
| J0039 | 340,420 | 115,562 | 115,562 | 114,162 | 113,812 | 113,812 | 110,545 |
| J0040 | 271,646 | 9,328 | 9,328 | 9,209 | 9,209 | 9,209 | 6,808 |
| J0050 | 332,095 | 104,637 | 104,637 | 28,728 | 28,665 | 28,665 | 24,678 |
| J0051 | 232,365 | 36,248 | 36,248 | 36,062 | 35,981 | 35,981 | 34,962 |
| J0052 | 357,353 | 250,965 | 250,965 | 249,034 | 237,689 | 237,689 | 235,442 |
| J0053 | 307,957 | 53,489 | 53,489 | 52,924 | 52,728 | 52,728 | 51,806 |
| J0054 | 250,136 | 121,093 | 121,093 | 109,341 | 105,044 | 105,044 | 46,909 |
| J0055 | 268,561 | 63,745 | 63,745 | 61,377 | 60,972 | 60,972 | 54,853 |
| J0056 | 332,451 | 77,011 | 77,011 | 74,208 | 73,873 | 73,873 | 70,477 |
| J0057 | 297,049 | 63,323 | 63,323 | 63,060 | 62,974 | 62,974 | 59,513 |
| J0058 | 335,288 | 236,809 | 236,809 | 236,517 | 236,382 | 236,382 | 234,660 |
| J0086 | 192,616 | 171,979 | 171,979 | 171,525 | 171,279 | 171,279 | 167,426 |
| J0088 | 1,491,929 | 1,383,306 | 1,383,306 | 1,379,037 | 1,336,894 | 1,336,894 | 1,331,599 |
| J0095 | 1,638,836 | 1,497,452 | 1,497,452 | 1,491,569 | 1,489,886 | 1,489,886 | 1,474,956 |
| J0098 | 232,310 | 143,432 | 143,432 | 142,127 | 136,478 | 136,478 | 3,734 |
| J0104 | 1,656,403 | 1,524,341 | 1,524,341 | 1,509,614 | 1,499,374 | 1,499,374 | 1,493,428 |
| J0105 | 1,546,867 | 1,399,077 | 1,399,077 | 1,378,762 | 1,376,993 | 1,376,993 | 1,327,934 |
| J0109 | 283,468 | 176,800 | 176,800 | 175,976 | 172,051 | 172,051 | 86,930 |
| J0110 | 1,886,593 | 1,748,296 | 1,748,296 | 1,733,227 | 1,627,024 | 1,627,024 | 869,572 |
| J0111 | 376,175 | 116,757 | 116,757 | 114,304 | 111,729 | 111,729 | 100,355 |
| J0113 | 1,519,119 | 1,320,445 | 1,320,445 | 1,301,786 | 1,236,830 | 1,236,830 | 1,234,519 |
| J0117 | 539,704 | 175,567 | 175,567 | 170,669 | 168,090 | 168,090 | 154,994 |
| J0119 | 1,774,664 | 1,635,459 | 1,635,459 | 1,612,483 | 1,611,219 | 1,611,219 | 1,605,933 |
| J0120 | 322,572 | 248,134 | 248,134 | 246,360 | 246,315 | 246,315 | 245,643 |
| J0121 | 353,262 | 251,633 | 251,633 | 248,289 | 248,284 | 248,284 | 245,806 |
| J0122 | 325,275 | 203,604 | 203,604 | 203,300 | 203,290 | 203,290 | 202,247 |
| J0124 | 1,858,198 | 1,708,300 | 1,708,300 | 1,689,381 | 1,688,149 | 1,688,149 | 1,688,038 |
| J0125 | 308,789 | 194,247 | 194,247 | 192,219 | 191,542 | 191,541 | 183,653 |
| J0127 | 1,747,212 | 1,565,574 | 1,565,574 | 1,554,288 | 1,553,818 | 1,553,818 | 1,553,529 |
| J0131 | 1,481,443 | 1,364,004 | 1,364,004 | 1,356,073 | 1,269,923 | 1,269,923 | 1,269,235 |
| J0133 | 1,700,803 | 1,457,693 | 1,457,693 | 1,439,026 | 1,418,968 | 1,418,968 | 628,485 |
| J0134 | 287,225 | 131,825 | 131,825 | 130,408 | 125,159 | 125,159 | 20,151 |
| J0136 | 313,162 | 193,011 | 193,011 | 160,985 | 160,414 | 160,414 | 150,561 |
| J0137 | 273,202 | 92,596 | 92,596 | 80,608 | 78,419 | 78,419 | 5,886 |
| J0138 | 284,324 | 103,165 | 103,165 | 95,876 | 91,177 | 91,177 | 8,251 |
| J0143 | 344,607 | 252,966 | 252,966 | 249,705 | 228,237 | 228,237 | 148,771 |
| J0144 | 378,690 | 86,599 | 86,599 | 84,285 | 83,211 | 83,211 | 62,530 |
| J0145 | 209,174 | 68,285 | 68,285 | 67,050 | 66,904 | 66,904 | 63,365 |
| J0146 | 290,848 | 164,276 | 164,276 | 163,397 | 161,140 | 161,140 | 156,590 |
| J0147 | 286,112 | 145,375 | 145,375 | 144,611 | 142,894 | 142,894 | 139,049 |
| J0149 | 417,876 | 320,888 | 320,888 | 314,129 | 299,728 | 299,728 | 284,540 |
| J0150 | 443,646 | 336,630 | 336,630 | 326,486 | 325,419 | 325,419 | 319,104 |
| J0151 | 405,168 | 339,433 | 339,433 | 332,998 | 332,148 | 332,148 | 320,991 |
| J0152 | 1,605,747 | 1,474,948 | 1,474,948 | 1,460,187 | 1,459,171 | 1,459,171 | 1,456,490 |
| J0153 | 407,070 | 333,290 | 333,290 | 326,374 | 325,667 | 325,667 | 319,440 |
| J0159 | 494,108 | 348,629 | 348,629 | 327,148 | 326,248 | 326,248 | 305,059 |
| J0160 | 1,286,219 | 1,194,655 | 1,194,655 | 1,184,711 | 1,086,797 | 1,086,797 | 1,083,185 |
| J0161 | 1,212,588 | 1,096,344 | 1,096,344 | 1,094,056 | 1,087,422 | 1,087,422 | 1,071,724 |
| J0162 | 387,664 | 305,319 | 305,319 | 302,047 | 291,536 | 291,536 | 289,961 |
| J0163 | 340,125 | 270,606 | 270,606 | 267,661 | 260,420 | 260,420 | 250,507 |
| J0164 | 1,100,839 | 939,087 | 939,087 | 927,930 | 911,002 | 911,002 | 906,026 |
| J0165 | 1,429,795 | 1,323,158 | 1,323,158 | 1,314,544 | 1,287,986 | 1,287,986 | 1,284,964 |
| J0166 | 1,499,621 | 1,387,100 | 1,387,100 | 1,379,136 | 1,354,116 | 1,354,116 | 1,352,455 |
| J0167 | 1,321,220 | 1,200,102 | 1,200,102 | 1,197,054 | 1,185,931 | 1,185,931 | 1,182,809 |
| J0168 | 1,447,924 | 1,233,903 | 1,233,903 | 1,224,640 | 1,191,896 | 1,191,896 | 1,190,946 |
| J0169 | 422,274 | 350,431 | 350,431 | 345,605 | 318,639 | 318,639 | 309,597 |
| J0170 | 438,445 | 351,747 | 351,747 | 345,824 | 342,404 | 342,404 | 338,910 |
| J0172 | 384,958 | 247,280 | 247,280 | 244,543 | 242,828 | 242,828 | 242,380 |
| J0175 | 443,460 | 260,593 | 260,593 | 254,791 | 254,163 | 254,163 | 240,156 |
| J0178 | 1,412,306 | 1,300,084 | 1,300,084 | 1,283,560 | 1,187,256 | 1,187,256 | 1,184,906 |
| J0180 | 1,143,200 | 955,442 | 955,442 | 940,701 | 870,977 | 870,977 | 868,697 |
| J0181 | 322,221 | 180,342 | 180,342 | 179,093 | 178,342 | 178,342 | 37,400 |
| J0184 | 457,561 | 347,154 | 347,154 | 344,197 | 343,970 | 343,970 | 342,961 |
| J0188 | 1,145,950 | 947,669 | 947,669 | 938,652 | 934,617 | 934,617 | 932,595 |
| J0189 | 1,051,718 | 961,009 | 961,009 | 951,528 | 872,937 | 872,937 | 871,651 |
| J0190 | 366,262 | 212,576 | 212,576 | 211,832 | 204,335 | 204,335 | 202,561 |
| J0191 | 1,087,946 | 931,712 | 931,712 | 920,689 | 917,961 | 917,961 | 916,871 |
| J0193 | 445,267 | 377,833 | 377,833 | 300,632 | 286,592 | 286,592 | 272,455 |
| J0195 | 1,255,696 | 1,155,533 | 1,155,533 | 1,141,236 | 1,140,393 | 1,140,393 | 1,137,899 |
| J0196 | 1,241,238 | 1,149,270 | 1,149,270 | 1,141,427 | 1,103,743 | 1,103,743 | 1,103,273 |
| J0199 | 1,217,328 | 1,075,128 | 1,075,128 | 1,064,867 | 996,926 | 996,926 | 995,522 |
| J0200 | 1,079,105 | 991,938 | 991,938 | 987,781 | 951,306 | 951,306 | 949,886 |
| J0202 | 388,387 | 298,948 | 298,948 | 297,338 | 295,685 | 295,685 | 253,548 |
| J0209 | 1,214,833 | 1,051,977 | 1,051,977 | 1,026,097 | 1,024,136 | 1,024,136 | 1,012,210 |
| J0216 | 427,258 | 332,516 | 332,516 | 198,980 | 194,931 | 194,931 | 140,253 |
| J0221 | 1,323,804 | 1,220,286 | 1,220,286 | 1,196,221 | 1,195,329 | 1,195,329 | 1,190,621 |
| J0225 | 1,572,769 | 1,453,612 | 1,453,612 | 1,441,088 | 1,318,668 | 1,318,668 | 1,314,373 |
| J0227 | 248,868 | 29,550 | 29,550 | 29,054 | 28,594 | 28,594 | 26,912 |
| J0228 | 1,235,382 | 1,110,781 | 1,110,781 | 1,101,042 | 1,004,328 | 1,004,328 | 1,001,652 |
| J0231 | 288,502 | 30,476 | 30,476 | 28,706 | 28,071 | 28,071 | 23,195 |
| J0234 | 347,979 | 69,757 | 69,757 | 65,811 | 64,477 | 64,477 | 28,739 |
| J0235 | 444,295 | 47,330 | 47,330 | 44,293 | 39,589 | 39,589 | 25,186 |
| J0237 | 304,053 | 40,467 | 40,467 | 39,874 | 39,408 | 39,408 | 25,709 |
| J0242 | 751,196 | 67,206 | 67,206 | 62,444 | 61,121 | 61,121 | 52,568 |
| J0246 | 417,169 | 328,111 | 328,111 | 323,892 | 323,420 | 323,420 | 318,215 |
| J0250 | 101,027 | 7,311 | 7,311 | 7,035 | 7,035 | 7,035 | 4,443 |
| J0260 | 1,555,545 | 1,450,769 | 1,450,769 | 1,439,120 | 1,327,413 | 1,327,413 | 1,325,124 |
| J0261 | 1,619,096 | 1,446,776 | 1,446,776 | 1,437,274 | 1,352,281 | 1,352,281 | 1,350,856 |
| J0262 | 306,091 | 205,023 | 205,023 | 200,352 | 196,929 | 196,929 | 186,919 |
| J0263 | 1,290,020 | 1,139,175 | 1,139,175 | 1,130,795 | 1,102,568 | 1,102,568 | 990,517 |
| J0267 | 273,169 | 153,175 | 153,175 | 151,621 | 151,328 | 151,328 | 150,751 |
| J0269 | 549,451 | 53,235 | 53,235 | 47,983 | 46,694 | 46,694 | 27,046 |
| J0271 | 195,449 | 73,626 | 73,626 | 73,517 | 72,700 | 72,700 | 72,119 |
| J0272 | 244,616 | 74,807 | 74,807 | 74,582 | 73,603 | 73,603 | 63,216 |
| J0274 | 156,704 | 61,731 | 61,731 | 61,114 | 60,336 | 60,336 | 56,527 |
| J0275 | 216,328 | 53,174 | 53,174 | 46,502 | 45,455 | 45,455 | 43,442 |
| J0276 | 1,610,930 | 1,478,045 | 1,478,045 | 1,472,615 | 1,437,549 | 1,437,549 | 1,434,620 |
| J0277 | 310,789 | 89,231 | 89,231 | 86,605 | 85,332 | 85,332 | 53,094 |
| J0278 | 305,034 | 116,106 | 116,106 | 85,825 | 84,008 | 84,008 | 18,755 |
| J0280 | 289,173 | 165,097 | 165,097 | 156,068 | 153,697 | 153,697 | 11,232 |
| J0281 | 393,048 | 315,768 | 315,768 | 311,739 | 310,271 | 310,271 | 301,909 |
| J0286 | 416,385 | 332,648 | 332,648 | 328,367 | 328,213 | 328,213 | 324,063 |
| J0287 | 379,395 | 295,291 | 295,291 | 292,932 | 292,759 | 292,759 | 291,655 |
| J0289 | 236,263 | 94,283 | 94,283 | 92,293 | 91,861 | 91,861 | 2,184 |
| J0300 | 350,741 | 270,484 | 270,484 | 265,578 | 264,976 | 264,975 | 253,234 |
| J0302 | 274,870 | 23,111 | 23,111 | 18,957 | 17,876 | 17,876 | 9,619 |
| J0303 | 285,605 | 120,041 | 120,041 | 106,694 | 103,300 | 103,300 | 3,112 |
| J0304 | 252,118 | 128,223 | 128,223 | 125,144 | 125,040 | 125,040 | 2,866 |
| J0306 | 1,232,345 | 1,026,460 | 1,026,460 | 1,012,792 | 1,004,554 | 1,004,554 | 37,641 |
| J0310 | 1,449,054 | 1,341,132 | 1,341,132 | 1,326,841 | 1,300,570 | 1,300,570 | 1,295,569 |
| J0312 | 313,439 | 266,440 | 266,440 | 256,941 | 256,924 | 256,924 | 256,451 |
| J0313 | 1,241,611 | 1,150,994 | 1,150,994 | 1,137,376 | 1,132,756 | 1,132,756 | 1,115,386 |
| J0314 | 242,653 | 171,462 | 171,462 | 171,187 | 171,135 | 171,135 | 169,048 |
| J0315 | 211,949 | 140,938 | 140,938 | 134,008 | 133,992 | 133,992 | 14,546 |
| J0316 | 381,904 | 327,884 | 327,884 | 324,987 | 324,987 | 324,987 | 323,698 |
| J0317 | 275,084 | 194,780 | 194,780 | 194,191 | 194,191 | 194,191 | 193,192 |
| J0321 | 1,479,928 | 1,355,507 | 1,355,507 | 1,332,152 | 1,329,866 | 1,329,866 | 1,327,013 |
| J0322 | 363,187 | 271,682 | 271,682 | 269,272 | 269,249 | 269,249 | 268,609 |
| J0339 | 1,042,990 | 963,435 | 963,435 | 953,241 | 951,700 | 951,700 | 951,459 |
| J0341 | 1,377,946 | 1,244,032 | 1,244,032 | 1,232,150 | 1,230,643 | 1,230,643 | 1,216,088 |
| J0342 | 429,222 | 328,395 | 328,395 | 323,885 | 322,351 | 322,351 | 321,304 |
| J0343 | 1,156,936 | 1,005,052 | 1,005,052 | 993,198 | 989,685 | 989,685 | 982,265 |
| J0347 | 1,180,231 | 1,096,507 | 1,096,507 | 1,089,888 | 995,567 | 995,567 | 994,109 |
| J0356 | 1,295,499 | 1,130,966 | 1,130,966 | 1,112,406 | 1,064,709 | 1,064,709 | 1,061,088 |
| J0359 | 1,149,756 | 976,486 | 976,486 | 970,028 | 906,353 | 906,353 | 896,555 |
| J0361 | 340,925 | 271,825 | 271,825 | 270,141 | 262,467 | 262,467 | 140,623 |
| J0362 | 1,225,402 | 1,093,868 | 1,093,868 | 1,089,287 | 1,088,145 | 1,088,145 | 1,086,859 |
| J0364 | 1,333,716 | 1,220,464 | 1,220,464 | 1,206,267 | 1,138,877 | 1,138,877 | 1,132,623 |
| J0368 | 1,372,878 | 1,162,766 | 1,162,766 | 1,141,825 | 1,138,217 | 1,138,217 | 1,135,392 |
| J0369 | 512,189 | 391,187 | 391,187 | 386,287 | 385,427 | 385,427 | 383,524 |
| J0371 | 521,550 | 435,257 | 435,257 | 431,184 | 429,355 | 429,355 | 426,461 |
| J0377 | 446,512 | 308,968 | 308,968 | 306,817 | 306,688 | 306,688 | 305,098 |
| J0379 | 483,092 | 388,435 | 388,435 | 382,207 | 377,478 | 377,478 | 348,544 |
| J0381 | 1,437,049 | 1,328,147 | 1,328,147 | 1,318,158 | 1,226,313 | 1,226,313 | 1,223,416 |
| J0385 | 539,906 | 447,451 | 447,451 | 445,082 | 437,883 | 437,883 | 365,690 |
| J0389 | 944,832 | 768,029 | 768,029 | 764,146 | 762,097 | 762,097 | 760,722 |
| J0390 | 1,411,672 | 1,316,540 | 1,316,540 | 1,313,590 | 1,303,443 | 1,303,443 | 1,301,110 |
| J0391 | 1,233,832 | 1,114,028 | 1,114,028 | 1,111,148 | 1,109,783 | 1,109,783 | 1,104,817 |
| J0398 | 1,253,493 | 1,134,548 | 1,134,548 | 1,130,846 | 1,086,661 | 1,086,661 | 1,086,155 |
| J0400 | 1,227,747 | 1,131,640 | 1,131,640 | 1,116,274 | 1,063,525 | 1,063,525 | 550,381 |
| J0403 | 420,320 | 346,947 | 346,947 | 342,348 | 342,317 | 342,317 | 340,380 |
| J0404 | 1,026,392 | 950,556 | 950,556 | 946,251 | 920,147 | 920,147 | 917,280 |
| J0406 | 1,593,145 | 1,401,541 | 1,401,541 | 1,381,027 | 1,298,410 | 1,298,410 | 1,292,820 |
| J0407 | 1,451,471 | 1,336,156 | 1,336,156 | 1,325,482 | 1,281,242 | 1,281,242 | 1,280,905 |
| J0408 | 309,058 | 203,237 | 203,237 | 202,550 | 201,432 | 201,432 | 197,714 |
| J0409 | 280,801 | 164,990 | 164,990 | 163,178 | 161,553 | 161,553 | 156,045 |
| J0414 | 1,918,694 | 1,711,788 | 1,711,788 | 1,695,322 | 1,629,961 | 1,629,961 | 1,620,561 |
| J0415 | 359,643 | 262,762 | 262,762 | 262,118 | 261,422 | 261,422 | 259,040 |
| J0416 | 2,486,673 | 2,107,599 | 2,107,599 | 1,853,617 | 1,828,243 | 1,828,243 | 1,493,542 |
| J0421 | 1,545,041 | 1,303,492 | 1,303,492 | 1,282,887 | 1,280,688 | 1,280,688 | 1,278,364 |
| J0423 | 1,537,039 | 1,371,437 | 1,371,437 | 1,350,703 | 1,348,726 | 1,348,726 | 1,346,275 |
| J0426 | 373,542 | 137,721 | 137,721 | 135,022 | 133,882 | 133,882 | 128,445 |
| J0431 | 1,341,872 | 1,072,069 | 1,072,069 | 1,050,586 | 1,035,774 | 1,035,774 | 1,027,864 |
| J0435 | 314,901 | 141,571 | 141,571 | 130,659 | 126,494 | 126,494 | 4,724 |
| J0439 | 389,214 | 287,709 | 287,709 | 285,842 | 272,582 | 272,582 | 265,978 |
| J0441 | 399,290 | 299,289 | 299,289 | 296,831 | 285,372 | 285,372 | 280,489 |
| J0442 | 276,082 | 188,950 | 188,950 | 180,505 | 179,479 | 179,479 | 131,302 |
| J0446 | 1,737,557 | 1,604,774 | 1,604,774 | 1,592,432 | 1,471,540 | 1,471,540 | 1,468,120 |
| J0447 | 1,601,618 | 1,312,039 | 1,312,039 | 1,292,789 | 1,283,418 | 1,283,418 | 1,279,351 |
| J0450 | 1,697,673 | 1,560,672 | 1,560,672 | 1,544,011 | 1,428,180 | 1,428,180 | 1,413,058 |
| J0455 | 1,863,314 | 1,725,203 | 1,725,203 | 1,706,726 | 1,703,610 | 1,703,610 | 1,695,993 |
| J0456 | 337,339 | 213,615 | 213,615 | 212,470 | 211,928 | 211,928 | 210,069 |
| J0459 | 298,297 | 204,480 | 204,480 | 200,238 | 196,169 | 196,169 | 117,313 |
| J0460 | 1,858,154 | 1,717,055 | 1,717,055 | 1,703,914 | 1,592,955 | 1,592,955 | 1,590,728 |
| J0462 | 1,758,375 | 1,553,340 | 1,553,340 | 1,526,689 | 1,437,338 | 1,437,338 | 1,358,068 |
| J0463 | 1,764,248 | 1,637,034 | 1,637,034 | 1,630,202 | 1,629,163 | 1,629,163 | 1,625,298 |
| J0466 | 362,555 | 292,423 | 292,423 | 283,274 | 280,189 | 280,189 | 275,091 |
| J0468 | 1,999,760 | 1,809,830 | 1,809,830 | 1,791,533 | 1,671,927 | 1,671,927 | 1,664,169 |
| J0469 | 432,527 | 287,424 | 287,424 | 284,263 | 282,927 | 282,927 | 276,622 |
| J0470 | 1,743,383 | 1,592,946 | 1,592,946 | 1,579,534 | 1,475,672 | 1,475,672 | 1,473,676 |
| J0471 | 299,288 | 223,103 | 223,103 | 221,127 | 217,963 | 217,963 | 124,771 |
| J0472 | 354,795 | 201,789 | 201,789 | 197,829 | 197,014 | 197,014 | 190,371 |
| J0473 | 238,333 | 89,918 | 89,918 | 85,973 | 85,904 | 85,904 | 78,295 |
| J0474 | 252,676 | 110,219 | 110,219 | 109,350 | 109,254 | 109,254 | 106,067 |
| J0475 | 316,568 | 154,255 | 154,255 | 149,989 | 149,940 | 149,940 | 142,697 |
| J0476 | 330,389 | 219,686 | 219,686 | 216,709 | 216,418 | 216,418 | 213,529 |
| J0477 | 338,544 | 268,669 | 268,669 | 263,111 | 253,155 | 253,155 | 251,190 |
| J0478 | 1,577,056 | 1,473,553 | 1,473,553 | 1,460,337 | 1,459,058 | 1,459,058 | 1,457,842 |
| J0479 | 299,112 | 239,608 | 239,608 | 238,094 | 237,954 | 237,954 | 237,290 |
| J0480 | 343,513 | 245,424 | 245,424 | 243,138 | 242,502 | 242,502 | 240,983 |
| J0481 | 307,873 | 224,967 | 224,967 | 222,538 | 217,213 | 217,213 | 215,361 |
| J0482 | 272,359 | 180,038 | 180,038 | 178,657 | 178,084 | 178,084 | 176,750 |
| J0483 | 267,597 | 101,893 | 101,893 | 95,850 | 93,891 | 93,891 | 80,423 |
| J0484 | 364,122 | 105,731 | 105,731 | 90,231 | 90,144 | 90,144 | 79,741 |
| J0485 | 307,851 | 77,318 | 77,318 | 76,283 | 76,140 | 76,140 | 71,783 |
| J0486 | 229,572 | 82,433 | 82,433 | 81,137 | 80,231 | 80,231 | 58,295 |
| J0487 | 280,890 | 91,822 | 91,822 | 81,495 | 81,110 | 81,110 | 68,809 |
| J0490 | 1,568,875 | 1,340,958 | 1,340,958 | 1,332,153 | 1,263,277 | 1,263,277 | 1,258,953 |
| J0491 | 313,430 | 154,693 | 154,693 | 153,895 | 153,820 | 153,820 | 152,963 |
| J0492 | 1,655,415 | 1,440,323 | 1,440,323 | 1,410,109 | 1,296,036 | 1,296,036 | 1,295,247 |
| J0494 | 507,818 | 99,867 | 99,867 | 95,772 | 94,718 | 94,718 | 86,998 |
| J0496 | 338,685 | 265,944 | 265,944 | 264,462 | 262,018 | 262,018 | 202,698 |
| J0497 | 318,995 | 188,090 | 188,090 | 185,672 | 183,145 | 183,145 | 3,719 |
| J0501 | 341,270 | 189,398 | 189,398 | 189,067 | 189,045 | 189,045 | 187,303 |
| J0502 | 274,988 | 130,599 | 130,599 | 129,889 | 129,463 | 129,463 | 2,782 |
| J0503 | 1,139,312 | 1,052,732 | 1,052,732 | 1,043,905 | 985,059 | 985,059 | 954,873 |
| J0504 | 356,097 | 251,417 | 251,417 | 248,412 | 243,807 | 243,807 | 242,292 |
| J0506 | 266,465 | 46,464 | 46,464 | 39,599 | 39,434 | 39,434 | 35,466 |
| J0507 | 1,154,036 | 983,386 | 983,386 | 969,677 | 966,904 | 966,904 | 965,346 |
| J0508 | 352,853 | 196,658 | 196,658 | 195,218 | 192,658 | 192,658 | 4,816 |
| J0512 | 286,629 | 79,479 | 79,479 | 78,773 | 78,747 | 78,747 | 76,810 |
| J0514 | 224,562 | 50,986 | 50,986 | 50,452 | 50,441 | 50,441 | 49,171 |
| J0515 | 389,368 | 271,095 | 271,095 | 268,131 | 268,121 | 268,121 | 266,426 |
| J0516 | 1,212,893 | 1,092,663 | 1,092,663 | 1,077,941 | 1,074,861 | 1,074,861 | 1,073,633 |
| J0517 | 293,511 | 71,040 | 71,040 | 69,523 | 69,410 | 69,410 | 67,480 |
| J0520 | 313,738 | 106,627 | 106,627 | 106,190 | 105,813 | 105,813 | 103,680 |
| J0522 | 296,057 | 96,577 | 96,577 | 95,187 | 95,155 | 95,155 | 94,206 |
| J0523 | 396,097 | 271,850 | 271,850 | 269,630 | 269,016 | 269,016 | 267,113 |
| J0524 | 1,061,573 | 960,304 | 960,304 | 947,034 | 943,250 | 943,250 | 942,556 |
| J0525 | 317,415 | 108,753 | 108,753 | 107,153 | 106,990 | 106,990 | 104,517 |
| J0528 | 831,102 | 767,651 | 767,651 | 764,527 | 762,516 | 762,516 | 751,134 |
| J0531 | 163,446 | 146,868 | 146,868 | 145,914 | 145,469 | 145,469 | 50,592 |
| J0555 | 394,710 | 370,990 | 370,990 | 369,720 | 352,719 | 352,719 | 348,652 |
| J0557 | 294,784 | 272,737 | 272,737 | 272,088 | 272,088 | 272,088 | 269,565 |
| J0565 | 230,642 | 217,253 | 217,253 | 217,068 | 217,066 | 217,066 | 216,352 |
| J0567 | 194,504 | 181,850 | 181,850 | 181,740 | 181,736 | 181,736 | 180,640 |
| J0574 | 255,601 | 234,352 | 234,352 | 233,637 | 221,277 | 221,277 | 219,876 |
| J0577 | 209,857 | 188,981 | 188,981 | 188,215 | 188,084 | 188,084 | 186,166 |
| J0582 | 1,081,301 | 1,002,685 | 1,002,685 | 999,008 | 961,854 | 961,854 | 959,352 |
| J0584 | 210,585 | 187,152 | 187,152 | 183,314 | 182,057 | 182,057 | 180,170 |
| J0587 | 257,929 | 206,052 | 206,052 | 205,895 | 205,895 | 205,895 | 205,068 |
| J0591 | 1,073,471 | 997,724 | 997,724 | 991,711 | 990,924 | 990,924 | 989,999 |
| J0595 | 1,042,691 | 923,106 | 923,106 | 907,084 | 906,445 | 906,445 | 906,244 |
| J0597 | 222,954 | 169,824 | 169,824 | 169,675 | 169,655 | 169,655 | 168,659 |
| J0600 | 870,163 | 779,608 | 779,608 | 769,505 | 751,145 | 751,145 | 749,924 |
| J0602 | 230,823 | 192,834 | 192,834 | 191,023 | 190,963 | 190,963 | 3,028 |
| J0608 | 1,094,207 | 1,014,556 | 1,014,556 | 1,001,289 | 925,221 | 925,221 | 923,279 |
| J0610 | 887,934 | 814,203 | 814,203 | 809,710 | 775,883 | 775,883 | 774,615 |
| J0611 | 254,899 | 208,186 | 208,186 | 207,301 | 205,370 | 205,370 | 204,594 |
| J0613 | 995,576 | 920,155 | 920,155 | 914,806 | 844,049 | 844,049 | 840,691 |
| J0615 | 375,179 | 330,054 | 330,054 | 326,200 | 326,103 | 326,103 | 324,603 |
| J0616 | 951,609 | 860,487 | 860,487 | 851,041 | 803,330 | 803,330 | 801,020 |
| J0624 | 1,176,144 | 1,091,374 | 1,091,374 | 1,085,156 | 997,928 | 997,928 | 995,488 |
| J0625 | 365,689 | 308,578 | 308,578 | 307,046 | 297,552 | 297,552 | 292,604 |
| J0630 | 303,831 | 248,648 | 248,648 | 248,451 | 248,423 | 248,423 | 247,505 |
| J0636 | 968,221 | 901,453 | 901,453 | 895,119 | 869,358 | 869,358 | 868,399 |
| J0638 | 1,198,337 | 1,098,905 | 1,098,905 | 1,093,821 | 1,070,126 | 1,070,126 | 1,069,957 |
| J0639 | 218,779 | 178,881 | 178,881 | 177,769 | 177,638 | 177,638 | 177,347 |
| J0647 | 1,394,513 | 1,176,796 | 1,176,796 | 1,167,912 | 1,161,482 | 1,161,482 | 1,160,646 |
| J0649 | 1,135,374 | 1,033,777 | 1,033,777 | 1,027,593 | 963,565 | 963,565 | 961,693 |
| J0650 | 1,036,118 | 918,254 | 918,254 | 914,255 | 912,828 | 912,828 | 909,404 |
| J0651 | 370,372 | 202,066 | 202,066 | 198,739 | 198,229 | 198,229 | 189,137 |
| J0654 | 487,115 | 262,305 | 262,305 | 258,780 | 257,734 | 257,734 | 250,793 |
| J0655 | 1,312,136 | 1,075,108 | 1,075,108 | 1,062,115 | 1,048,526 | 1,048,526 | 1,042,152 |
| J0660 | 1,091,127 | 912,451 | 912,451 | 894,041 | 892,142 | 892,141 | 884,696 |
| J0662 | 347,522 | 145,206 | 145,206 | 142,896 | 133,786 | 133,786 | 14,483 |
| J0665 | 1,086,106 | 992,403 | 992,403 | 984,956 | 984,410 | 984,410 | 983,829 |
| J0666 | 1,166,262 | 1,084,037 | 1,084,037 | 1,079,264 | 1,012,658 | 1,012,658 | 1,012,465 |
| J0668 | 1,318,254 | 1,088,718 | 1,088,718 | 1,081,334 | 1,064,065 | 1,064,065 | 1,062,364 |
| J0670 | 374,171 | 213,175 | 213,175 | 211,304 | 210,823 | 210,823 | 208,500 |
| J0674 | 1,106,803 | 1,010,398 | 1,010,398 | 999,075 | 929,465 | 929,465 | 220,514 |
| J0678 | 325,733 | 235,272 | 235,272 | 233,003 | 231,905 | 231,905 | 96,750 |
| J0679 | 430,474 | 345,138 | 345,138 | 342,738 | 323,843 | 323,843 | 322,351 |
| J0682 | 356,009 | 255,187 | 255,187 | 253,937 | 253,927 | 253,927 | 251,965 |
| J0683 | 1,115,752 | 1,021,138 | 1,021,138 | 1,002,369 | 973,608 | 973,608 | 972,557 |
| J0686 | 1,093,434 | 999,489 | 999,489 | 996,537 | 958,429 | 958,429 | 955,847 |
| J0688 | 1,337,815 | 1,163,959 | 1,163,959 | 1,149,109 | 1,098,980 | 1,098,980 | 1,092,156 |
| J0692 | 1,142,257 | 932,343 | 932,343 | 918,570 | 916,475 | 916,475 | 895,176 |
| J0695 | 521,348 | 231,518 | 231,518 | 230,002 | 229,579 | 229,579 | 2,642 |
| J0696 | 840,857 | 27,967 | 27,967 | 17,369 | 16,209 | 16,209 | 3,537 |
| J0697 | 352,686 | 180,160 | 180,160 | 178,947 | 178,706 | 178,706 | 177,825 |
| J0698 | 343,521 | 202,059 | 202,059 | 201,345 | 201,142 | 201,142 | 198,165 |
| J0700 | 411,390 | 274,047 | 274,047 | 270,068 | 262,312 | 262,312 | 261,114 |
| J0702 | 1,749,825 | 1,529,285 | 1,529,285 | 1,512,080 | 1,405,924 | 1,405,924 | 1,227,663 |
| J0703 | 1,080,107 | 931,658 | 931,658 | 920,104 | 879,574 | 879,574 | 859,947 |
| J0706 | 1,304,029 | 1,193,946 | 1,193,946 | 1,181,064 | 1,120,989 | 1,120,989 | 1,082,270 |
| J0713 | 1,462,351 | 1,252,432 | 1,252,432 | 1,233,910 | 1,192,180 | 1,192,180 | 1,190,237 |
| J0717 | 1,441,456 | 1,191,825 | 1,191,825 | 1,172,225 | 1,105,570 | 1,105,570 | 403,716 |
| J0720 | 1,497,667 | 1,288,577 | 1,288,577 | 1,252,889 | 1,160,430 | 1,160,430 | 29,235 |
| J0721 | 1,578,724 | 1,416,485 | 1,416,485 | 1,398,884 | 1,296,927 | 1,296,927 | 973,429 |
| J0723 | 320,725 | 238,013 | 238,013 | 234,953 | 226,299 | 226,299 | 5,558 |
| J0724 | 450,324 | 355,213 | 355,213 | 338,198 | 303,532 | 303,532 | 3,134 |
| J0725 | 439,838 | 318,357 | 318,357 | 310,248 | 289,505 | 289,505 | 2,375 |
| J0727 | 1,462,320 | 1,282,745 | 1,282,745 | 1,260,451 | 1,196,238 | 1,196,238 | 836,088 |
| J0728 | 385,617 | 140,685 | 140,685 | 135,182 | 127,330 | 127,330 | 8,158 |
| J0729 | 1,490,330 | 1,362,410 | 1,362,410 | 1,345,744 | 1,310,354 | 1,310,354 | 881,923 |
| J0730 | 300,187 | 156,519 | 156,519 | 154,203 | 150,366 | 150,366 | 68,529 |
| J0731 | 312,331 | 220,458 | 220,458 | 217,296 | 212,769 | 212,769 | 205,113 |
| J0733 | 373,545 | 235,278 | 235,278 | 231,427 | 231,152 | 231,152 | 227,631 |
| J0735 | 1,353,277 | 1,158,566 | 1,158,566 | 1,157,079 | 1,156,529 | 1,156,529 | 1,155,213 |
| J0736 | 1,305,243 | 1,138,362 | 1,138,362 | 1,132,413 | 1,074,015 | 1,074,015 | 1,072,465 |
| J0756 | 369,396 | 168,799 | 168,799 | 167,560 | 164,568 | 164,568 | 161,858 |
| J0759 | 1,658,222 | 1,544,272 | 1,544,272 | 1,533,298 | 1,523,854 | 1,523,854 | 1,517,727 |
| J0761 | 1,342,601 | 1,188,333 | 1,188,333 | 1,165,095 | 1,161,755 | 1,161,755 | 1,157,306 |
| J0765 | 1,445,285 | 1,237,413 | 1,237,413 | 1,209,165 | 1,207,362 | 1,207,362 | 1,203,458 |
| J0766 | 258,686 | 185,027 | 185,027 | 183,726 | 175,731 | 175,731 | 174,911 |
| J0767 | 257,130 | 79,601 | 79,601 | 76,953 | 76,027 | 76,027 | 68,677 |
| J0768 | 283,814 | 107,846 | 107,846 | 103,633 | 102,764 | 102,764 | 93,247 |
| J0769 | 323,626 | 126,552 | 126,552 | 92,324 | 90,849 | 90,849 | 70,102 |
| J0770 | 250,023 | 105,148 | 105,148 | 90,544 | 89,587 | 89,587 | 84,105 |
| J0771 | 336,934 | 210,813 | 210,813 | 208,488 | 207,462 | 207,462 | 193,003 |
| J0772 | 292,029 | 89,141 | 89,141 | 87,818 | 87,129 | 87,129 | 83,649 |
| J0779 | 235,125 | 110,133 | 110,133 | 109,238 | 109,238 | 109,238 | 103,158 |
| J0783 | 1,304,362 | 1,113,937 | 1,113,937 | 1,097,629 | 1,085,665 | 1,085,665 | 1,084,279 |
| J0785 | 318,885 | 220,789 | 220,789 | 211,508 | 205,364 | 205,364 | 13,902 |
| J0786 | 226,233 | 103,112 | 103,112 | 102,145 | 100,463 | 100,463 | 29,225 |
| J0787 | 281,294 | 85,240 | 85,240 | 81,096 | 79,079 | 79,079 | 16,083 |
| J0789 | 1,998,745 | 1,835,360 | 1,835,360 | 1,814,996 | 1,810,567 | 1,810,567 | 1,765,419 |
| J0790 | 380,704 | 303,315 | 303,315 | 283,448 | 279,319 | 279,319 | 160,583 |
| J0792 | 236,856 | 165,508 | 165,508 | 163,658 | 163,653 | 163,653 | 162,819 |
| J0793 | 260,092 | 144,774 | 144,774 | 141,506 | 141,103 | 141,103 | 137,296 |
| J0794 | 266,436 | 209,580 | 209,580 | 191,561 | 191,279 | 191,279 | 185,610 |
| J0795 | 327,498 | 209,722 | 209,722 | 201,570 | 197,486 | 197,486 | 6,875 |
| J0796 | 274,401 | 211,191 | 211,191 | 209,669 | 207,888 | 207,888 | 7,005 |
| J0800 | 199,512 | 101,372 | 101,372 | 97,988 | 97,119 | 97,119 | 92,375 |
| J0801 | 1,809,167 | 1,685,404 | 1,685,404 | 1,665,508 | 1,567,183 | 1,567,183 | 1,560,432 |
| J0802 | 241,682 | 167,739 | 167,739 | 134,853 | 133,796 | 133,796 | 115,187 |
| J0806 | 2,079,406 | 1,880,159 | 1,880,159 | 1,840,888 | 1,836,674 | 1,836,674 | 1,756,468 |
| J0807 | 208,109 | 77,864 | 77,864 | 76,514 | 76,071 | 76,071 | 38,631 |
| J0815 | 1,377,826 | 1,141,870 | 1,141,870 | 1,125,270 | 1,056,145 | 1,056,145 | 1,052,820 |
| J0816 | 305,154 | 163,853 | 163,853 | 157,642 | 155,367 | 155,367 | 6,109 |
| J0818 | 1,702,299 | 1,526,846 | 1,526,846 | 1,490,734 | 1,475,245 | 1,475,245 | 690,380 |
| J0819 | 328,746 | 237,196 | 237,196 | 226,007 | 224,672 | 224,672 | 207,564 |
| J0821 | 1,897,781 | 1,723,749 | 1,723,749 | 1,694,545 | 1,693,850 | 1,693,850 | 1,678,807 |
| J0822 | 317,746 | 231,469 | 231,469 | 229,055 | 227,058 | 227,058 | 110,861 |
| J0823 | 1,449,911 | 1,269,194 | 1,269,194 | 1,239,328 | 1,236,428 | 1,236,428 | 1,228,076 |
| J0827 | 1,339,729 | 1,098,463 | 1,098,463 | 1,085,333 | 1,084,166 | 1,084,166 | 1,080,795 |
| J0831 | 362,267 | 24,256 | 24,256 | 22,271 | 21,818 | 21,817 | 15,093 |
| J0833 | 1,358,197 | 1,234,991 | 1,234,991 | 1,227,269 | 1,219,476 | 1,219,476 | 1,211,635 |
| J0836 | 252,922 | 67,591 | 67,591 | 67,194 | 66,872 | 66,872 | 61,855 |
| J0843 | 1,231,301 | 1,134,249 | 1,134,249 | 1,131,017 | 1,078,867 | 1,078,867 | 1,077,932 |
| J0845 | 430,902 | 128,125 | 128,125 | 126,790 | 125,988 | 125,988 | 122,574 |
| J0846 | 1,465,978 | 1,313,594 | 1,313,594 | 1,311,103 | 1,309,366 | 1,309,366 | 1,308,150 |
| J0848 | 425,846 | 281,568 | 281,568 | 278,470 | 267,492 | 267,492 | 265,574 |
| J0849 | 1,711,682 | 1,480,624 | 1,480,624 | 1,467,417 | 1,358,170 | 1,358,170 | 1,353,818 |
| J0850 | 309,114 | 9,710 | 9,710 | 9,597 | 9,222 | 9,222 | 6,076 |
| J0851 | 309,306 | 5,209 | 5,209 | 4,904 | 4,786 | 4,786 | 3,269 |
| J0854 | 303,050 | 11,961 | 11,961 | 11,608 | 11,309 | 11,309 | 6,171 |
| J0860 | 492,814 | 49,395 | 49,395 | 47,141 | 46,291 | 46,291 | 39,993 |
| J0862 | 129,869 | 3,164 | 3,164 | 2,972 | 2,970 | 2,970 | 2,452 |
| J0866 | 67,973 | 5,938 | 5,938 | 5,665 | 5,620 | 5,620 | 2,726 |
| J0868 | 110,576 | 8,049 | 8,049 | 7,562 | 7,517 | 7,517 | 6,529 |
| J0871 | 794,370 | 8,496 | 8,496 | 7,126 | 7,103 | 7,103 | 5,528 |
| J0873 | 182,515 | 12,013 | 12,013 | 11,124 | 11,124 | 11,124 | 7,955 |
| J0875 | 292,785 | 223,164 | 223,164 | 220,657 | 217,836 | 217,836 | 5,042 |
| J0877 | 1,263,407 | 1,152,612 | 1,152,612 | 1,148,379 | 1,147,495 | 1,147,495 | 1,145,010 |
| J0878 | 246,518 | 47,422 | 47,422 | 46,847 | 46,792 | 46,792 | 44,615 |
| J0882 | 284,931 | 167,482 | 167,482 | 167,252 | 167,248 | 167,248 | 165,012 |
| J0883 | 284,681 | 169,957 | 169,957 | 169,479 | 168,649 | 168,649 | 3,663 |
| J0884 | 293,769 | 90,818 | 90,818 | 90,408 | 89,757 | 89,757 | 4,965 |
| J0885 | 386,934 | 263,968 | 263,968 | 262,268 | 262,265 | 262,265 | 261,658 |
| J0889 | 82,283 | 14,646 | 14,646 | 14,505 | 14,505 | 14,505 | 13,427 |
| J0891 | 213,029 | 18,795 | 18,795 | 10,470 | 10,469 | 10,469 | 3,534 |
| J0894 | 159,026 | 4,690 | 4,690 | 4,473 | 4,473 | 4,473 | 3,746 |
| J0895 | 292,188 | 156,256 | 156,256 | 156,019 | 154,879 | 154,879 | 3,818 |
| J0904 | 98,131 | 10,562 | 10,562 | 10,315 | 10,279 | 10,279 | 9,787 |
| J0905 | 76,763 | 10,052 | 10,052 | 9,963 | 9,636 | 9,636 | 9,043 |
| J0907 | 276,071 | 117,305 | 117,305 | 115,309 | 112,239 | 112,239 | 110,500 |
| J0908 | 1,413,500 | 1,303,177 | 1,303,177 | 1,289,064 | 1,185,094 | 1,185,094 | 1,178,572 |

**Supplementary Table S5.** Overview of the 16S rRNA gene sequencing dataset for the negative control generated using HiSeq.

| Sample | Input | Quality filtered | Denoised | Non-chimeric |
| --- | --- | --- | --- | --- |
| *Negative1 - forward | 214 | 22 | 22 | 22 |
| Negative1 - reverse | 214 | 0 | 0 | 0 |
| Negative2 - forward | 1,800 | 168 | 168 | 168 |
| Negative2 - reverse | 1,800 | 2 | 2 | 2 |
| Negative3 - forward | 1,447 | 121 | 121 | 121 |
| Negative3 - reverse | 1,447 | 8 | 8 | 8 |

* “Blank” negative DNA extraction/PCR controls (i.e., PCR products of template acquired from a sham extraction to which no faecal sample was added).

**Supplementary Table S6.** Taxonomic assignment data for the negative control.

| ASV ID | The number of reads | | | Taxonomy  (SILVA 132 database) |
| --- | --- | --- | --- | --- |
|  | *Negative1 | Negative2 | Negative3 |  |
| 4e0dda61787f456f9390bccb13908927 | 22 | 0 | 0 | Unassigned |
| 1b223fddd21cb3b2d1b09516941d49c6 | 0 | 168 | 0 | Unassigned |
| 4389fea9be1625bc0a500c64bc7ee8d5 | 0 | 0 | 121 | Unassigned |
| 50fd40bed4c597db0ac989afce9b852d | 0 | 2 | 0 | D_0__Bacteria;__;__;__;__;__;__ |
| 3846d08a335948c79bdc3f9fa21b05b9 | 0 | 0 | 8 | D_0__Bacteria;__;__;__;__;__;__ |

* “Blank” negative DNA extraction/PCR controls (i.e., PCR products of template acquired from a sham extraction to which no faecal sample was added).

**References**

1. Raskin L, Stromley JM, Rittmann BE, Stahl DA. Group-specific 16S rRNA hybridization probes to describe natural communities of methanogens**.** Appl Environ Microbiol. 1994;60**:**1232-40.

2. Stahl D, Amann R: Development and application of nucleic acid probes. In Nucleic acid techniques in bacterial systematics. Chichester, England: John Wiley & Sons Ltd.; 1991. p. 205-48.

3. Takai K, Horikoshi K. Rapid detection and quantification of members of the archaeal community by quantitative PCR using fluorogenic probes**.** Appl Environ Microbiol. 2000;66**:**5066-72.

4. Ovreas L, Forney L, Daae FL, Torsvik V. Distribution of bacterioplankton in meromictic Lake Saelenvannet, as determined by denaturing gradient gel electrophoresis of PCR-amplified gene fragments coding for 16S rRNA**.** Appl Environ Microbiol. 1997;63**:**3367-73.

5. Ritalahti KM, Amos BK, Sung Y, Wu Q, Koenigsberg SS, Loffler FE. Quantitative PCR targeting 16S rRNA and reductive dehalogenase genes simultaneously monitors multiple *Dehalococcoides* strains**.** Appl Environ Microbiol. 2006;72**:**2765-74.

6. Amann RI, Binder BJ, Olson RJ, Chisholm SW, Devereux R, Stahl DA. Combination of 16S rRNA-targeted oligonucleotide probes with flow cytometry for analyzing mixed microbial populations**.** Appl Environ Microbiol. 1990;56**:**1919-25.
